# Effect of chimeric antigen receptor T cells against protease-activated receptor 1 for treating pancreatic cancer

**DOI:** 10.1186/s12916-023-03053-9

**Published:** 2023-09-04

**Authors:** Hao-Chien Hung, Ming-Huei Fan, Daniel Wang, Carol H. Miao, Pong Su, Chao-Lien Liu

**Affiliations:** 1 Department of General Surgery, Chang-Gung Memorial Hospital at Linkou, Taoyuan, 33305 Taiwan; 2 School of Medical Laboratory Science and Biotechnology, College of Medical Science and Technology, Taipei Medical University, 250 Wu-Hsing Street, Taipei, 11031 Taiwan; 3 Center for Immunity and Immunotherapies, Seattle Children’s Research Institute, Seattle, WA 98101 USA; 4 Ph.D. Program in Medical Biotechnology, College of Medical Science and Technology, Taipei Medical University, Taipei, 11031 Taiwan

**Keywords:** Pancreatic ductal adenocarcinoma (PDAC), Pancreatic cancer, Protease-activated receptor 1 (PAR1), Chimeric antigen receptor (CAR), TGF-β

## Abstract

**Background:**

Pancreatic ductal adenocarcinoma (PDAC) is a devastating malignancy with a 5-year survival rate of 6% following a diagnosis, and novel therapeutic modalities are needed. Protease-activated receptor 1 (PAR1) is abundantly overexpressed by both tumor cells and multiple stroma cell subsets in the tumor microenvironment (TME), thereby offering a suitable immunotherapy target.

**Methods:**

A chimeric antigen receptor (CAR) strategy was applied to target PAR1 using a human anti-PAR1 scFv antibody fused to the transmembrane region with two co-stimulatory intracellular signaling domains of cluster of differentiation 28 (CD28) and CD137 (4-1BB), added to CD3ζ in tandem.

**Results:**

The engineered PAR1CAR-T cells eliminated PAR1 overexpression and transforming growth factor (TGF)-β-mediated PAR1-upregulated cancer cells by approximately 80% in vitro. The adoptive transfer of PAR1CAR-T cells was persistently enhanced and induced the specific regression of established MIA PaCa-2 cancer cells by > 80% in xenograft models. Accordingly, proinflammatory cytokines/chemokines increased in CAR-T-cell-treated mouse sera, whereas Ki67 expression in tumors decreased. Furthermore, the targeted elimination of PAR1-expressing tumors reduced matrix metalloproteinase 1 (MMP1) levels, suggesting that the blocking of the PAR1/MMP1 pathway constitutes a new therapeutic option for PDAC treatment.

**Conclusions:**

Third-generation PAR1CAR-T cells have antitumor activity in the TME, providing innovative CAR-T-cell immunotherapy against PDAC.

**Supplementary Information:**

The online version contains supplementary material available at 10.1186/s12916-023-03053-9.

## Background

Pancreatic ductal adenocarcinoma (PDAC) is a fatal malignancy of both the digestive system and endocrine system with disappointing prognoses. The number of newly diagnosed PDAC cases worldwide is approaching five million each year. As the seventh leading cause of cancer-related deaths, PDAC also accounts for more than 4.6 million new deaths [1]. Given the increase in the incidence of PDAC, it is estimated that PDAC will surpass breast cancer as the third leading cause of cancer death by 2025 [2]. Currently, standard therapy for patients with PDAC focuses on conventional chemotherapeutic regimens (e.g., gemcitabine, oxaliplatin, and FORLFIRINOX) [3] and curative-intent surgical resection if possible; yet, outcomes are disappointing with a 5-year survival rate of 6% [4]. The few choices of therapeutic options with limited effects, the advanced stage at presentation due to late detection, and the vicious behavior of PDAC contribute to the high mortality rate [5]. Therefore, developing novel therapies for PDAC is direly needed.

Protease-activated receptor 1 (PAR1), a cell surface 7-transmembrane G protein-coupled receptor, is irreversibly activated by its agonists, including thrombin, tissue factor (TF), endothelial protein C receptor, and matrix metalloproteases (MMPs) [6]. PAR1 is expressed by many cell subsets, including epithelial cells, neurons, immune cells, myocytes, and astrocytes, and PAR1 also participates in multiple molecular mechanisms of biological functions [7–9]. PAR1 overexpression was reported in various types of malignancies, including mammary, renal, gastric, colorectal, lung, pancreatic, esophageal, prostate, hepatic, and ovarian cancers [10–17]. Abundant PAR1 overexpression promotes PDAC tumorigenesis, fibroblast activation, extracellular matrix (ECM) production, and cytokine secretion in the tumor microenvironment (TME), and it is present not only on tumor cells but also in multiple dominant components of stromal cell subsets, such as endothelial cells, cancer-associated fibroblasts (CAFs), and tumor-associated macrophages (TAMs) [18]. These processes indicate a negative correlation with survival in patients with PDAC [13, 19]. Accordingly, reports demonstrated that alternatively spliced TF (asTF) binds to β1 integrins on the surface of PDAC cells and the ligated asTF significantly increases expressions of three essential cell adhesion molecules (CAMs) required for adhesion between leukocytes and the endothelium in the stroma, namely endothelial E-selectin, vascular cell adhesion protein (VCAM)-1, and intercellular cell adhesion molecule (ICAM)-1. The asTF-β1-integrin interaction initiates downstream signaling and induces endothelial cell migration, thus promoting tumor growth and metastasis [20–22]. Additionally, the thrombin-PAR1 pathway activates nuclear Ca^2+^ signaling and facilitates pancreatic stellate cell proliferation, contributing to the development of PDAC [23]. PAR1 promotes PDAC progression by being implicated in suppressing antitumor immunity via regulation of coagulation cascades and host immune responses [24]. Given the numerous deleterious effects of PAR1 signaling in PDAC, the PAR1 pathway may serve as an attractive target for PDAC immunotherapy and may be indicated for future translational applications [25].

Chimeric antigen receptor (CAR)-T cells [26], also known as living drugs, are powerful immunotherapeutic agents that are capable of recognizing and binding to specific circulating antigens or tumor targets independent of the major histocompatibility complex (MHC) without tissue-type restraints. CAR-T cells are engineered immune cells to treat cancer that have great potentiality as immunotherapeutic remedies for some of the most difficult cancers to treat, such as PDAC. Currently, the main targets of preclinical trials using CAR-engineered T cells to treat PDAC are carcinoembryonic antigen (CEA), human epidermal growth factor receptor 2 (HER2), mucin 1 (MUC1), prostate stem cell antigen, prominin 1 (PROM1), EGFR, and mesothelin (MSN) [27, 28]. Although CAR-T-cell therapy has been used against several targets (e.g., CEA and HER2) to treat PDAC [29], most of these target candidates are tumor-associated antigens (TAAs) with shared expressions in both malignant and healthy tissues [30–34]. Furthermore, CAR-T-cell toxicity in humans has garnered some attention [35, 36], indicating that optimal CARs for novel targets have yet to be determined.

In this study, we revealed a successful antitumor strategy with CAR-T cells against PAR1-expressing PDAC cells, in which the CAR was effective and safe in both in vitro and PDAC xenograft murine models. The specificity of PAR1-based (PAR1CAR) engineering is directly derived from the scFv region of the human αPAR1-specific monoclonal antibody (mAb), and has a substantial effect on CAR-T cells recognizing and binding to TAAs. Consistently, engineered PAR1CAR-T cells efficiently recognized and eliminated approximately 80% of human PDAC cells in vitro and suppressed the tumorigenesis of PDAC xenografts.

Transforming growth factor (TGF)-β-mediated PAR1 upregulation enhanced PDAC cell responsiveness to PAR1CAR-T-cell-specific attack, indicating an expanded cytotoxic effect of PAR1CAR-T cells toward tumor cells even if the cancer cell exhibited low or no PAR1 expression. Mechanistic studies revealed that PAR1CAR-T cells produced cytokines (e.g., tumor necrosis factor (TNF)-α and interferon (IFN)-γ) upon stimulation with PAR1^+^ tumor cells and acquired potent cytolytic activity against PAR1^+^ tumor cells. Additionally, the targeted elimination of PAR1-expressing tumors reduced the level of matrix metalloproteinase 1 (MMP1), which acts as a tumorigenesis promoter. This suggests that blocking of the MMP1-PAR1 signaling pathway may represent a new therapeutic option for PDAC. Together, our results provide proof of principle with encouraging evidence for the development of PAR1-targeting CAR-T-cell therapy for treating PDAC.

## Methods

### Ethics, consent, and permission

This study (protocol no.: N201605059; June 10, 2016) was approved by the Taipei Medical University-Joint Institutional Review Board (TMU-JIRB). The study protocol (e.g., the use of biohazards, biological agents, toxins, materials, and reagents) followed standard biosafety regulations and was reviewed and approved by the Institute’s Environmental Protection and Biological Safety Committee (G-104–078; January 4, 2016) before the study commenced.

### Cell lines

Human PDAC cell lines (MIA PaCa-2, SU.8686, HPAF-II, Capan-1, ASPC-1, and CFPAC-1), normal human cell lines (WS1, Hs181.Tes, MRC-5, and Hs67), and 293 T cells were purchased from American Type Culture Collection (ATCC; Manassas, VA, USA) and grown in Dulbecco’s modified Eagle’s medium (DMEM; Life Technologies, Carlsbad, CA, USA) supplemented with 10% fetal bovine serum (FBS) at 37 °C with 5% CO_2_ incubation. Species identification, mycoplasma detection, and authentication were confirmed by ATCC using short tandem repeat profile analyses for all cell lines.

### Construction of a lentiviral vector encoding the CAR

**Fig. 1:**
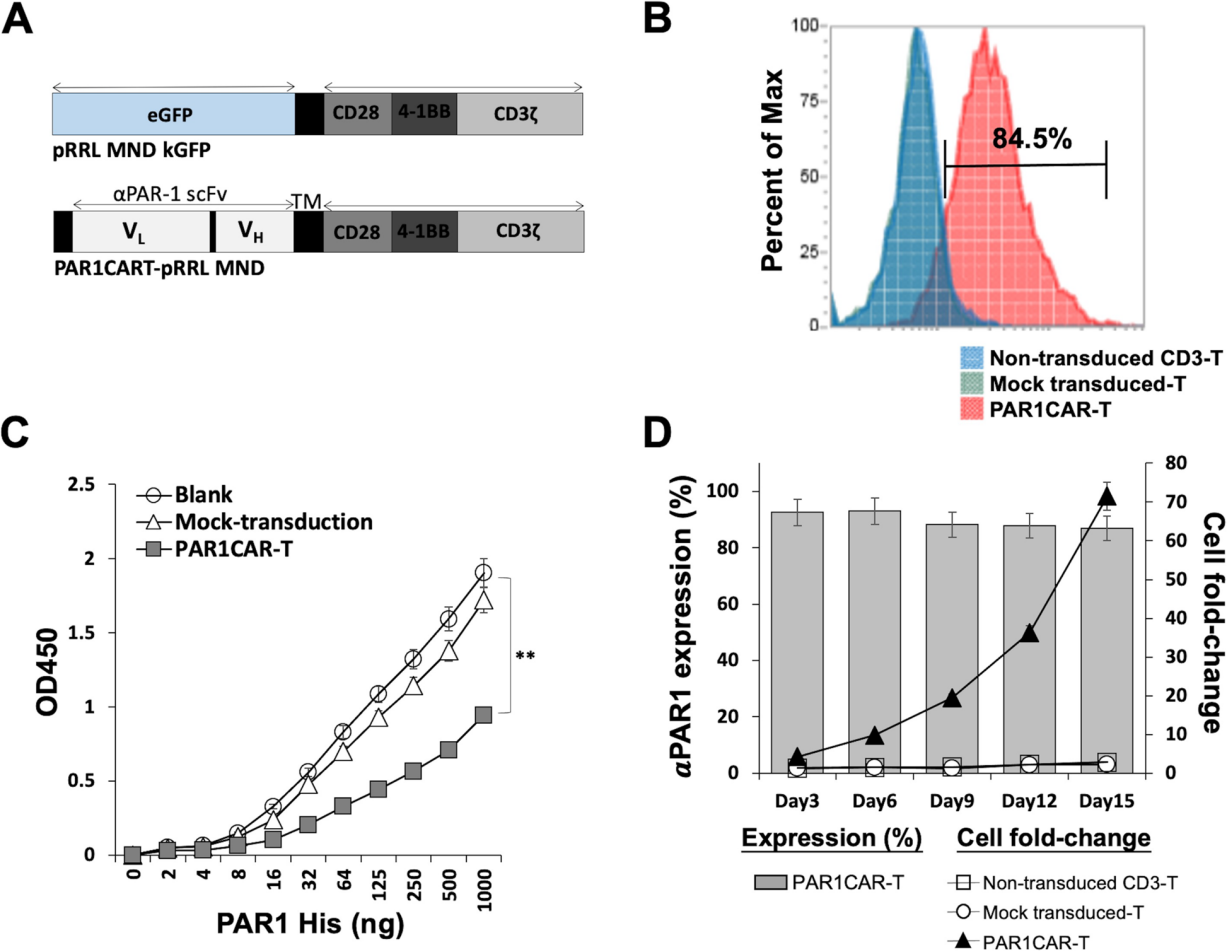
Characterization of genetically engineered PAR1CAR-T cells. **A** Schematic of a lentiviral vector (LV) expressing a chimeric antigen receptor (CAR). **B** Anti-PAR1 expression levels in human T cells transduced with LV particles were analyzed using flow cytometry by detecting expression of an αPAR1 antibody. Transduction efficiencies are plotted in histograms. The 84.5% transduction rate is not an average but an individual result that served as a minimum requirement in our laboratory protocol. **C** A sandwich ELISA was performed to evaluate the binding ability of the PAR1CAR-T PAR1 antigen using PAR1CAR- and mock-transduced 293 T cells. Non-transduced 293 T cells were employed as a negative control (blank; ** *p* < 0.01). **D** PAR1CAR-T cell expression percentages are plotted as bars, and cell fold-changes are expressed in line plots, compared to mock-transduced T cells and non-transduced CD3 T cells. Data were derived from experiments involving three independent healthy donors during a 2-week culture period to stimulate the recombinant human PAR1-His.Tag protein

The nucleotide sequence encoding the human anti-PAR1 (αPAR1) scFv Ab in the V_L_-V_H_ orientation was codon optimized and synthesized (Life Technologies). The shuttle plasmid pRRLMNDkGFP lentiviral vector (LV) was used to construct PAR1CAR. As indicated in Fig. 1A, a third-generation αPAR1CAR comprising the scFv PAR1 linked in-frame to the hinge domain of a cluster of differentiation 8α (CD8α) molecule (GenBank: NM_001145873.1) was fused to the transmembrane (TM) region and intracellular signaling domains of the human CD28 (GenBank: NM_006139.3) and CD137 molecules (4-1BB; GenBank: NM_001561.5), and added to CD3ζ (GenBank: NM_198053.2) in tandem.

### Lentivirus production and the transduction of human CD3 T cells

In total, 9 × 10^6^ human 293 T cells were seeded into each 15-cm dish prior to 24 h of transfection. All plasmid DNA was purified using an EndoFree Maxi prep kit (Qiagen, Valencia, CA, USA). 293 T cells were transfected with an empty vector (as the mock control) or a recombinant expression vector using a calcium phosphate transduction system [37], and then the viral supernatant was harvested 48 h after transduction in accordance with the protocol described in our previous report [38]. Peripheral blood mononuclear cells (PBMCs) were derived from healthy human donors. Primary human CD3 T cells were then isolated from PBMCs by positive selection using a REAlease CD3 MicroBead kit (Miltenyi Biotec, Bergisch Gladbach, Germany), and isolated CD3 T cells without activation served as non-transduced CD3 T cells used in further assays. Instead of using isolated CD3 T cells stored for more than 72 h, we routinely prepared cells and double-confirmed the cell viability before each experimental course. Isolated CD3 T cells were then stimulated with recombinant human IL-2 (100 U/mL; PeproTech, city?, NJ, USA) plus anti-CD3 antibodies (10 ng/mL; eBioscience, Pittsburgh, PA, USA) for 24 h; and then transduced with the LV at a multiplicity of infection (MOI) of 16 U/cell under the approach described in our previous report [ref?]. Transduced CD3 T cells were cultured in DMEM (Life Technologies) with 10% FBS and 1% L-glutamine at 8 × 10^5^ cells/mL in the presence of recombinant human IL-2 (300 IU/mL) every other day. Genetically modified T cells were isolated using a flow sorter (FACSAria III; BD Bioscience, San Jose, CA, USA) before being used for functional assays.

### Sandwich enzyme-Linked Immunosorbent Assay (ELISA)

To evaluate the binding ability of the PAR1CAR-To PAR1 antigen, a sandwich ELISA was performed following an earlier description [39]. In brief, 96-well plates were seeded with transduced 293 T cells (PAR1CAR and mock control). Untransduced 293 T cells (blank) were used as a negative control. Various dilutions of recombinant human PAR1-His antigens (Sino Biological, Wayne, PA, USA) were added to each well. Subsequently, supernatants were collected and added to another 96-well plate, which was precoated with an anti-His.Tag (horseradish peroxidase; HRP) MAb (0.2 μg/mL; Sino Biological). After substrate detection, the optical density at 450 nm (OD450) was measured with an automatic microplate reader (BioTek, Winooski, VT, USA).

### Real-time reverse-transcription quantitative polymerase chain reaction (RT-qPCR)

Cancer cells were cultured, collected, and lysed for total RNA extraction using a LabPrep RNA Plus Mini Kit (Taigen Bioscience, Taipei, Taiwan). Complementary (c)DNA was synthesized through reverse transcription of RNA using a High-Capacity cDNA Reverse Transcription Kit (Thermo Fisher Scientific, Foster City, CA, USA) according to the manufacturer’s instructions. A qPCR was performed in triplicate for each cDNA sample on an Applied Biosystems StepOne and StepOnePlus Real-Time PCR analyzer (Applied Biosystems, Waltham, USA) using PowerUp SYBR Green Master Mix (Thermo Fisher Scientific). Normalization was performed with the *S26* gene according to the crossing threshold (Ct) value of the transcripts assessed using a real-time qPCR. Changes in messenger (m)RNA expression levels are expressed as multiples of change relative to the control ± standard deviation (SD). The primer sequences used were as follows:

PAR1-forward (F): 5′-CGTTTAGTGAACCGTCAGAT-3′; PAR1-reverse (R): 5′-GGAGTTATTGATCCTCACAA-3′; TF-F: 5′-AAGCACTAAGTCAGGAGATTGG-3′; TF-R: 5′-AACCGGTGCTCTCCACATTCCC-3′; thrombin-F: 5′-AAGCACGGTCGCTGCTCC-3′; thrombin R: 5′-TTGGCCCAGAACACATCC-3′

S26-F: 5′-CCGTGCCTCCAAGATGACAAAG-3′; and S26-R: 5′-GTTCGGTCCTTGCGGGCTTCAC-3′.

### Western blot analysis

For the Western blot analysis, cells were harvested and lysed in protein lysis buffer (Merck Millipore, St. Louis, MO, USA) supplemented with a complete protease inhibitor cocktail. Cell lysates were prepared in a previously described manner [40]. Primary antibodies against PAR1 (ATAP2:sc-13503; Santa Cruz Biotechnology, Dallas, TX, USA), RhoA, ROCK1, glyceraldehyde-3-phosphate dehydrogenase (GAPDH) (Cell Signaling, Danvers, MA, USA), and β-actin were purchased from Novus Biologicals (Littleton, CO, USA), both GAPDH and β-actin were used as loading controls. Appropriate HRP-conjugated secondary antibodies were used to detect proteins using a luminol-based enhanced chemiluminescent (ECL) substrate (Thermo Scientific). An ImageQuant LAS 4000 analyzer (GE Healthcare Life Science, Pittsburgh, PA, USA) was used to detect protein expression levels.

### Flow cytometry

CAR-modified T cells and PDAC cell lines were prepared using previously described standard protocols. A human PAR1 (thrombin R or ATAP2) phycoerythrin (PE)-conjugated antibody (sc-13503 PE; Santa Cruz Biotechnology), mAb, was used for PDAC cell line detection. For CAR-T-cell detection, recombinant human PAR1 (human F2R-His Tag, cat. no: 13535-H08H; Sino Biological) was conjugated. Subsequently, an APC-anti-His.Tag antibody (BioLegend, San Diego, CA, USA) and its immunoglobulin G (IgG) controls were conjugated against recombinant human PAR1 for detection. An Annexin V/PI (propidium iodide) Apoptosis Detection Kit (BD Biosciences) was used for apoptotic cell examination. A flow cytometric assay was performed using a FACSCanto II flow cytometer, and results were analyzed using CellQuest Pro (BD Biosciences) software.

### Immunocytofluorescence (IF)

Cells were seeded in eight-well chamber slides (Thermo Fisher Scientific) and cultured for 48 h at 37 °C with 5% CO_2_. Serum starvation was conducted for 24 h, and the aforementioned cells were fixed with 4% paraformaldehyde for 10 min at room temperature. Subsequently, cells were permeabilized in 0.4% Triton-X for 10 min and blocked in 3% bovine serum albumin (BSA) in phosphate-buffered saline (PBS) for 1 h. Following three washes with PBS, cells were incubated with a primary Ab, namely antihuman PAR1 (ATAP2: sc-13503; Santa Cruz Biotechnology), for 2 h. Primary Abs were washed off with PBS, and cells were incubated with a 1:100 dilution of secondary Abs, namely mouse IgGκ light chain-binding protein conjugated to fluorescein isothiocyanate (m-IgGκ BP-FITC: sc-516140; Santa Cruz Biotechnology), for another 2 h. Coverslips were mounted onto each slide using two drops of mounting medium containing 4,6-diamidino-2-phenylindole (DAPI), a highly sensitive nucleic acid stain. Fluorescence images were acquired using a BX51 Olympus fluorescence microscope (Tokyo, Japan).

### 3-(4,5-Dimethylthiazol-2-yl)-2,5-diphenyltetrazolium bromide (MTT) cytotoxicity assays

In 96-well plates, MTT (Sigma-Aldrich, Saint Louis, MO, USA) cytotoxicity assays were performed. Tumor cells (10^4^ cells/well) were plated in triplicate wells. After 24 h, the indicated titration ratios of PAR1CAR-T cells, mock-transduced T cells, and non-transduced CD3 T cells at approximate effector/tumor (E/T) ratios were added for another 24 h. After incubation, the plate was washed twice with PBS to remove the cell supernatant before reading the MTT reduction. MTT values were evaluated at an absorbance of 570 nm with an ELISA reader (BioTek). Cell viability was calculated as a percentage of MTT reduction.

### Real-time life cell monitoring assay

The xCELLigence system was used to monitor cell survival according to the supplier’s instructions (Roche Diagnostics, Mannheim, Germany and ACEA Bioscience, San Diego, CA, USA). Cells were grown overnight with impedance measured every hour prior to treatments as described in a previous study [41]. Cell impedance was measured using the cell index [CI = (Zi − Z0) ohms/15 ohms, where Zi is the impedance at an individual time point and Z0 is the background resistance]. A normalized CI is determined as the CI at a certain time point divided by the CI at the normalization time point.

### In vivo studies

Nude mice (BALB/cAnN.Cg-Foxn1nu/CrlNarl) aged 6 to 8 weeks were maintained in a specific pathogen-free facility according to National Institutes of Health guidelines for animal care and guidelines of Taipei Medical University (Taipei, Taiwan). All animal experiments were performed according to protocols reviewed and approved by the Institutional Animal Care and Use Committee/Institutional Animal Care and Use Program (IACUC/IACUP; protocol no.: LAC-2019–0091, March 12, 2019). For established subcutaneous (s.c.) MIA PaCa-2 models, mice were s.c. inoculated with 10^7^ MIA PaCa-2 cells in the left flank on day 0. Mice were randomly assigned to one of four treatment groups (*n* = 6 mice/group) as follows: (i) Ctrl (1 × PBS) only without T cells, (ii) non-transduced CD3 T cells in 1 × PBS, (iii) mock-transduced T cells in 1 × PBS (Mock), and (iv) genetically modified PAR1CAR-T cells in 1 × PBS at 10^6^ cells/mouse delivered through an intravenous injection. All measurements were executed using calipers across mice skins to gauge the tumor size rather than radiological imaging, each week after tumor cells were implanted in the mice. As of the ninth-week measurement, none of our mice met the standard to be sacrificed, either in terms of mental status, appetite, vitality, body weight, hair, or tumor size. However, in the last measurement before the scheduled sacrifice in the tenth week, there were indeed several mice (*n* = 4) whose tumors exceeded the specification of size regulation with no limitation of physical activities. After dissection at week 10, tumor xenografts were first measured (the largest size was 2.9 cm) and then fixed with formalin. Since the time for the end of the experiment had also been reached, these estimates were used for calculation and analysis. Tumor volumes were calculated with the following formula: *V* = 1/2 (length × width^2^). At the endpoint dissection, xenografts were measured, fixed with formalin, embedded in paraffin, and processed for immunohistochemical (IHC) staining. Mice that died during the experimental period were excluded.

### Cytokine assay

Mouse serum cytokine profiles were assessed using the Human Cytokine/Chemokine Magnetic Bead Panel protocol for the Milliplex Map Kit (cat. no. HCYTOMAG-60 K; Millipore, Billerica, MA, USA). In brief, assay plates were washed and mixed for 10 min at room temperature. After the addition of samples or controls, samples were incubated overnight at 4 °C on an orbital shaker with fluorescently labeled capture antibody-coated beads, which were used for the simultaneous quantification of the following 41 human cytokines and chemokines in serum samples according to the manufacturer’s recommendations: epidermal growth factor (EGF), eotaxin, granulocyte colony-stimulating factor (G-CSF), granulocyte–macrophage (GM)-CSF, interferon (IFN)-alpha 2, IFN-γ, interleukin (IL)-10, IL-12P70, IL-13, IL-15, IL-17A, IL-1RA, IL-1α, IL-1β, IL-2, IL-3, IL-4, IL-5, IL-6, IL-7, IL-8, IP-10, monocyte chemotactic protein (MCP)-1, macrophage inflammatory protein (MIP)-1α, MIP-1β, regulated upon activation normal T cell expressed and secreted (RANTES), TNF-α, TNF-β, vascular endothelial growth factor (VEGF), fibroblast growth factor (FGF)-2, TGF-α, farnesyl transferase inhibitor (FIT)-3L, fractalkine, growth-regulated oncogene (GRO), MCP-3, macrophage-derived chemokine, platelet-derived growth factor (PDGF)-AA, PDGF-AB/BB, sCD40L, and IL-9. Plates were run on the Luminex MagPix machine, and data were collected using Luminex xPONENT software (vers. 4.2). Cytokine and chemokine levels were analyzed using Milliplex Analyst software (vers. 5.1).

### IHC analysis and immunoreactivity scoring

For the IHC analysis, serial tumor Sects. (4 μm thick) were prepared from formalin-fixed tumor samples and mounted on glass slides. Sample sections were stained with mAbs against human Ki67, CD3, MMP1, and PAR1; hematoxylin counterstaining was performed after 10 min of 3,3’-diaminobenzidine incubation. Images were captured using a BX50 Olympus microscope (Tokyo, Japan). For semiquantitative analysis of marker immunoreactivity, the H-score was used as previously described [42]. In brief, at least 10 fields were counted in each case, and the H-score was subsequently generated by adding the percentages of strongly stained (3 ×), moderately stained (2 ×), and weakly stained (1 ×) cells, giving a possible range of 0 − 300.

### Statistical analysis

All data in the figures are presented as the mean ± SD of three independent experiments. Statistical analysis was performed for intergroup comparisons using two-tailed Student’s *t* test. Comparisons between groups were conducted using a one-way analysis of variance. Differences were considered significant at *p* < 0.05; statistical analyses were performed using IBM SPSS® vers. 24.0 (SPSS, Chicago, IL, USA).

## Results

### Generation of PAR-specific CAR-T cells through LV transduction

To generate PAR1CAR-T cells, the human immunodeficiency virus (HIV)-based lentiviral plasmid pRRLMNDkGFP (pMND-Neo; Cell Biolabs, San Diego, CA, USA) (Fig. 1A, upper panel) was used to form the construction. A PAR1CAR LV (Fig. 1A, lower panel) with a high package efficiency of approximately 5 × 10^9^ IFU/mL was developed (data not shown). To develop the PAR1CAR LV, a sequence encoding the anti-PAR1 scFv Ab in the VL-VH orientation comprising scFv PAR1 linked to the transmembrane domain was fused to the intracellular signaling domains derived from the CD3ζ, 4-1BB (CD137), and CD28 molecules. CAR expression of transduced T cells was demonstrated by recognizing the recombinant human PAR1-His.Tag protein, which is conjugated to the APC-anti-His.Tag Ab. In a flow cytometric (Attune NxT Flow Cytometer; Life Technologies, Carlsbad, CA, USA) analysis, an 84.5% transduction efficiency of PAR1CAR was found (Fig. 1B), which was significantly higher than that of the mock-transduced T cell and non-transduced CD3 T cell controls. αPAR1-specific CAR expression levels were analyzed by flow cytometry. Transduction efficiency rates are shown in Additional file 1: Figure S1. To elucidate the affinity of the scFv component of engineered CAR-T cells toward the target antigen (PAR1), we transduced 293 T cells with PAR1CAR, and used LV transduction cells and 293 T cells without transduction as mock and negative controls, respectively (Fig. 1C). Binding titration curves of PAR1CAR-T cells were plotted by the mean of OD450 readings against recombinant human PAR1 His antigen concentration levels, while mock-transduced T cells and non-transduced 293 T cells (blank; Sino Biological, Wayne, PA, USA) served as the control groups. Results clearly proved good binding of PAR1CAR-T cells to the PAR1 His antigen (** *p* < 0.01; Fig. 1C), indicating that the scFv fragment of the redirected PAR1CAR-T cell demonstrated functional PAR1 antigen binding. To further investigate the proliferative ability of the genetically engineered T cells, activated CAR-T cells were cultured in complete medium in the presence of 0.75 μg/mL of the recombinant human PAR1 His protein, compared to mock-transduced T cells and non-transduced CD3 T cells. Cell counts and expression levels of CAR-T cells were measured at several time points for up to 15 days (Fig. 1D). Anti-PAR1 Ab-expressing CAR-T cells increased by over 70-fold following PAR1 antigen stimulation. Additionally, the transduction efficiency was excellent with the surface expression frequency of CAR on the T cells maintained at high levels (at approximately 80%) throughout the entire expansion period (Fig. 1D).

### Abundant expression of PAR1 by human PDAC cells

**Fig. 2:**
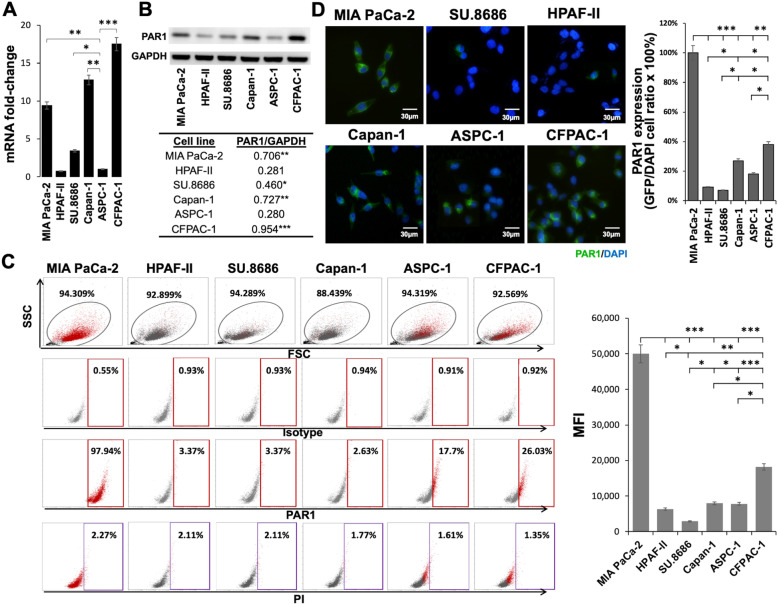
Endogenous PAR1 expression in different human pancreatic ductal adenocarcinoma (PDAC) cells. Six human PDAC cell lines, namely MIA PaCa-2, HPAF-II, SU.8686, Capan-1, ASPC-1, and CFPAC-1, were cultured for PAR1 level screening. **A** RT-PCR analysis of endogenous PAR1 mRNA levels (*** *p* < 0.001). **B** Western blot analysis of PAR1 expression (~ 66 kDa) in whole-cell lysates among the six cell lines (*** *p* < 0.001). **C** The mean fluorescence intensity (MFI) of PAR1 surface expression by tumor cells was determined by a flow cytometric analysis using a phycoerythrin (PE)-anti-PAR1 antibody (Ab) versus an isotype control. Propidium iodide (PI) levels were used to examine apoptotic cells. **D** Immunocytofluorescence (IF) analysis of PAR1 expression patterns in PDAC cells using antihuman PAR1 with signal enhancement through m-IgGκ BP-FITC labeling (left panel). Quantified statistics of green fluorescent protein-positive (GFP^+^) to DAPI.^+^ cell ratio of IF results are also shown (right panel). Individual scale bars are shown. All data are presented as the mean ± SD. of three experiments. *** *p* < 0.001

To investigate the efficacy of CAR-T-cell-related therapeutic strategies against PAR1-expressing PDAC-TME, PAR1 mRNA and protein expression levels in six PaC cell lines (MIA PaCa-2, HPAF-II, SU.8686, Capan-1, ASPC-1, and CFPAC-1) were determined through RT-qPCR, Western blot, flow cytometric, and IF analyses. mRNA expressions were significantly higher in MIA PaCa-2, Capan-1, and CFPAC-1 cells (Fig. 2A) than in the other cell lines. Comparisons of relative PAR1 expression levels of mRNA and whole-cell lysates were validated with the use of a real-time PCR (Fig. 2A) and Western blotting (Fig. 2B) in the six PDAC cell lines. Results of the two tests were similar in that significant expression was observed in the MIA PaCa-2, Capan-1, and CFPAC1 cell lines. According to flow cytometric analyses among all cell lines (Fig. 2C), the highest PAR1 level on the surface was expressed by MIA PaCa-2 cells (with an MFI of 49,995), followed by the CFPAC-1 cells (with an MFI of 18,195). We also provided statistical details regarding the GFP^+^ to DAPI^+^ cell ratio of IF results (Fig. 2D, right panel). The quantified percentages of PAR1 expression were consistent with the quantitative flow cytometric data (Fig. 2C). As the flow cytometric immunophenotyping results indicated, MIA PaCa-2 cells revealed the most abundant surface PAR1 expression, compared to other cells (** *p* < 0.01 and *** *p* < 0.001). Based on different surface expression levels of PAR1 by the six representative PDAC cell lines, we chose the following three cell lines for further investigation: MIA PaCa-2 (high PAR1 expression), CFPAC-1 (medium PAR1 expression), and HPAF-II (low PAR1 expression).

### In vitro cytotoxic activity of PAR1CAR-T cells toward PAR1-expressing human PDAC cells

**Fig. 3:**
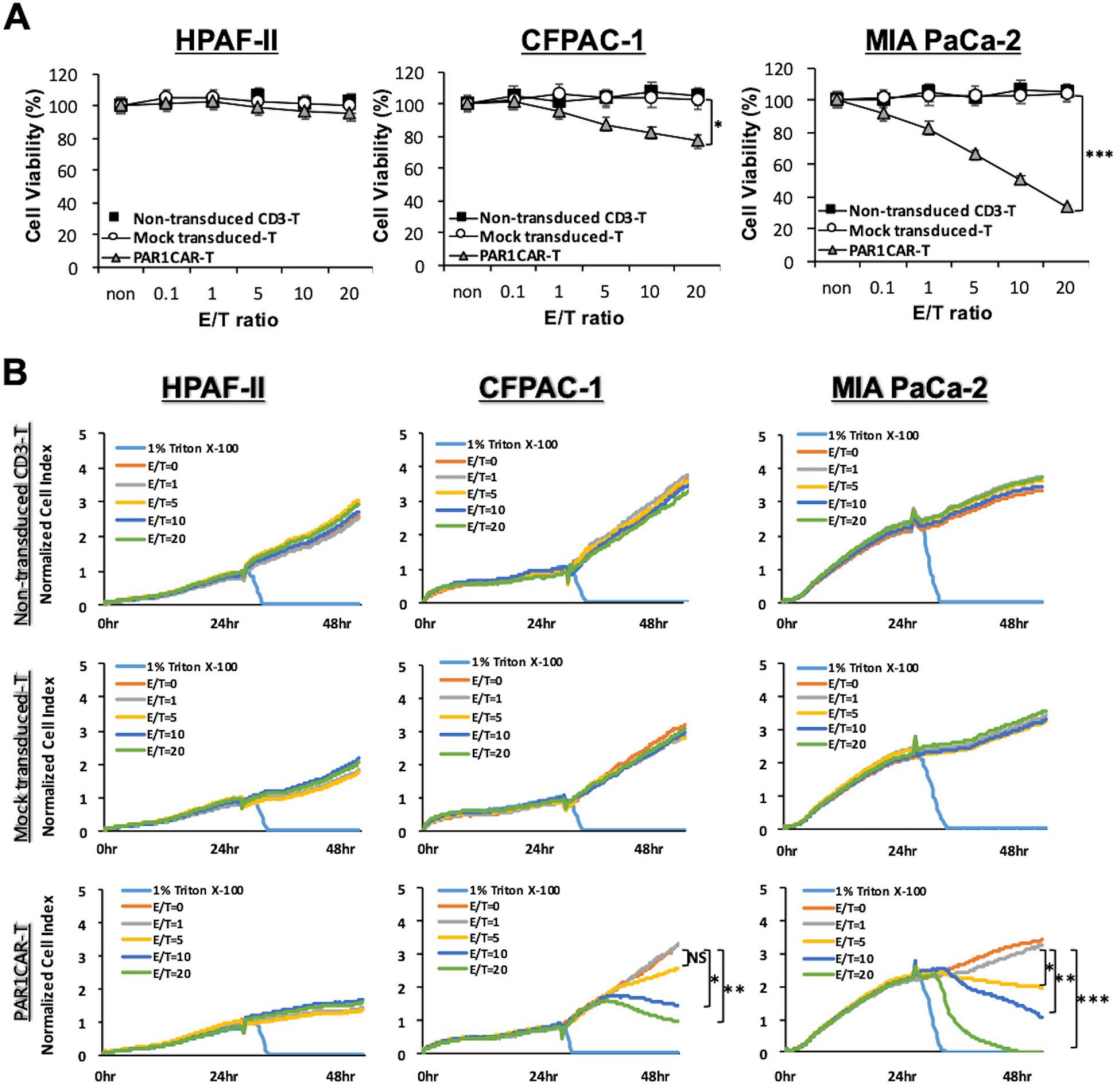
Suppression of PAR1-expressing MIA PaCa-2 and CFPAC-1 cells by PAR1CAR-T cells in vitro. **A** A standard 24-h MTT cytotoxicity assay using three replicates (*n* > 3) with increasing effector/tumor (E/T; effector: PAR1CAR-T cells) ratios of 0, 0.1, 1, 5, 10, and 20 against pancreatic ductal adenocarcinoma (PDAC) cell lines of MIA PaCa-2, CFPAC-1, and HPAF-II. Cytotoxic activities were compared to those of non-transduced CD3^+^ T-cell-treated cells, and mock-transduced T-cell-treated cells served as the control PAR1CAR-T cells (*n* > 3; * *p* < 0.05 and *** *p* < 0.001). **B** Real-time monitoring of cytotoxic activities used for comparison between non-transduced CD3^+^ T cell-treated and mock-transduced T cell-treated cells, and 1% Triton-X-100-treated cells served as a positive control. Real-time monitoring of PAR1CAR-T-cell-treated cells revealed specific growth inhibition of PAR1-expressing CFPAC-1 (low levels; *n* > 3; * *p* < 0.05 and ** *p* < 0.01) and MIA PaCa-2 cells (high levels; *n* > 3; * *p* < 0.05, ** *p* < 0.01, and *** *p* < 0.001) compared to PAR1 non-expressing HPAF-II cells, as observed using the x-CELLigence System. Data are presented as the mean ± SD of three independent experiments

To confirm whether PAR1CAR-T cells can specifically recognize and eliminate PAR1-expressing tumor cells, in vitro cytotoxicity assays were performed by incubating PAR1CAR-T cells with MIA PaCa-2, CFPAC-1, and HPAF-II cells at E/T ratios of 0.1, 1, 5, 10, and 20. PAR1CAR-T cells efficiently triggered cell lysis of PAR1-high MIA PaCa-2 (*** *p* < 0.001) and PAR1-medium CFPAC-1 (* *p* < 0.05) cells but not PAR1-low HPAF-II cells, as observed by MTT assays (Fig. 3A). In contrast, neither of the control effector cells, mock-transduced T cells, nor non-transduced CD3 T cells could initiate cell lysis (Fig. 3A). PAR1CAR-T cell-mediated cytotoxicity was assessed using an impedance-based real-time cell analyzer, the xCELLigence system [43]. In the first 24 h, tumor cells (MIA PaCa-2, CFPAC-1, and HPAF-II) grew proportionally over the culture time. The addition of PAR1CAR-T cells led to an abrupt decrement in impedance with E/T ratios of 10 and 20 for PAR1-medium CFPAC-1 cells and with E/T ratios of 5, 10, and 20 for PAR1-high MIA PaCa-2 cells, reflecting tumor cell death. The cell index (CI) values of PAR1-medium CFPAC-1 cells and PAR1-high MIA PaCa-2 cells also significantly moved in a downward direction, while the CI value of PAR1-low HPAF-II cells continued to grow after adding PAR1CAR-T cells (Fig. 3B). CI values of all three cell lines treated with corresponding E/T ratios of mock-transduced and non-transduced CD3 T cells steadily increased over time as expected (Fig. 3B). These results corresponded to the MTT assays (Fig. 3A), indicating that our engineered PAR1CAR-T cells acquired designated cytotoxic activity toward PAR1-enriched tumor cells.

### TGF-β-mediated PAR1 upregulation enhances PDAC cell responsiveness to PAR1CAR-T cell-specific targeting

A study revealed that TGF-β increased PAR1 gene, protein, and cell surface expressions by A549 cells, thereby promoting lung cancer progression [44]. To further improve the cytotoxicity of PAR1CAR-T cells against tumor cells with lower PAR1 expression, we first treated six PDAC cell lines, namely CFPAC-1, Capan-1, ASPC-1, SU.8686, HPAF-II, and MIA PaCa-2, with 1 ng/mL TGF-β and examined their cell surface PAR1 levels by flow cytometry at indicated time points within 48 h (Additional file 2: Figure S2). CFPAC-1 cells initially exhibited relatively lower expression levels of endogenous PAR1, with an initial corresponding MFI of 14,791 (Additional file 2: Figure S2A, left panel). Exposure to TGF-β (1 ng/mL) led to a time-dependent upregulation of cell surface PAR1 MFI levels (with a post-treatment absolute MFI of 36,257), and it reached a nearly 2.5-fold increase after 48 h (Additional file 2: Figure S2A, right panel). After 48 h of TGF-β stimulation, enhanced cytotoxic activity of PAR1CAR-T cells in TGF-β-mediated PAR1-upregulated CFPAC-1 cells was observed (Additional file 2: Figure S2C), compared to unexposed CFPAC-1 cells with only medium endogenous PAR1 levels which induced less cytotoxic activity (Fig. 3A, middle panel); results were determined through MTT assays.

**Fig. 4:**
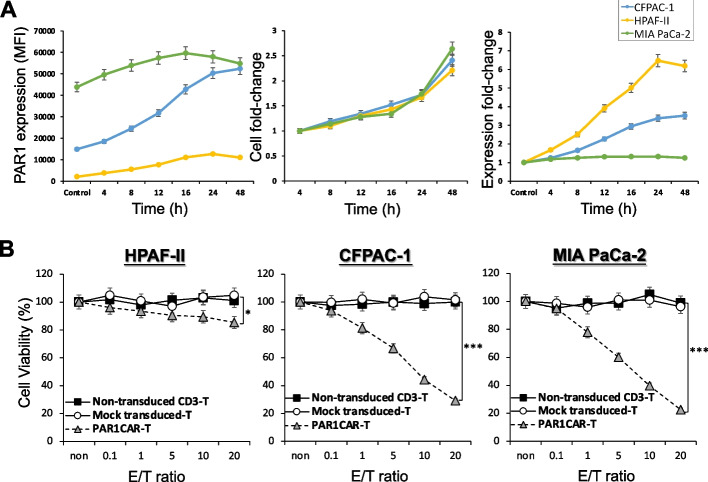
Transforming growth factor (TGF)-β-mediated PAR1 upregulation enhances pancreatic ductal adenocarcinoma (PDAC) cell responsiveness to PAR1CAR-T-cell-specific suppression. **A** Human PDAC cell lines (HPAF-II, CFPAC-1, and MIA PaCa-2) were exposed to TGF-β (18 ng/mL), and cells were collected at indicated times over 48 h. PAR1 expression was measured by flow cytometry. Results revealed original and enhanced levels of PAR1 by quantifying the mean fluorescent intensity (MFI) (left panel), expression fold-changes (right panel), and cell fold-changes (middle panel) over incubation times. **B** Standard 24-h cytotoxic activities of PAR1CAR-T cells toward tumor cells were measured using MTT assays with increasing effector/tumor (E/T) ratios of 0, 0.1, 1, 5, 10, and 20 against HPAF-II, CFPAC-1, and MIA PaCa-2 cells following 18 ng/mL TGF-β stimulation (18 ng/mL) for 48 h. Cytotoxic activities were compared to those of non-transduced CD3^+^ T-cell-treated cells, and mock-transduced T-cell-treated cells served as control PAR1CAR-T cells (*n* > 3; * *p* < 0.05 and *** *p* < 0.001, respectively). Results are the mean ± SD of three independent experiments

The effects of TGF-β stimulation on tumor cells were both time and dosage dependent. Endogenous PAR1 expression was at a low MFI level by HPAF-II cells and a medium level by CFPAC-1 cells. Exposure to a high level of TGF-β (18 ng/mL) for 48 h led to 5.3- and 3.5-fold increases in expression in HPAF-II (with the absolute MFI changing from 2068 to 11,030) and CFPAC-1 cells (with the absolute MFI changing from 14,891 to 52,393), respectively (Fig. 4A, left and right panel). Significantly increased cytotoxic activity of PAR1CAR-T cells toward TGF-β-mediated PAR1-upregulated HPAF-II and CFPAC-1 cells was observed (Fig. 4B, * *p* < 0.05 and *** *p* < 0.001, respectively), compared to unexposed HPAF-II and CFPAC-1 cells with lower levels of endogenous PAR1.Taken together, as a positive regulator of PAR-1 expression, TGF-β stimulation subsequently enhanced tumor cell responsiveness to PAR1CAR-T-cell-specific targeting.

### CAR-T cells redirected to PAR1 significantly suppress tumorigenesis in the TME of subcutaneous MIA PaCa-2 xenografts

**Fig. 5:**
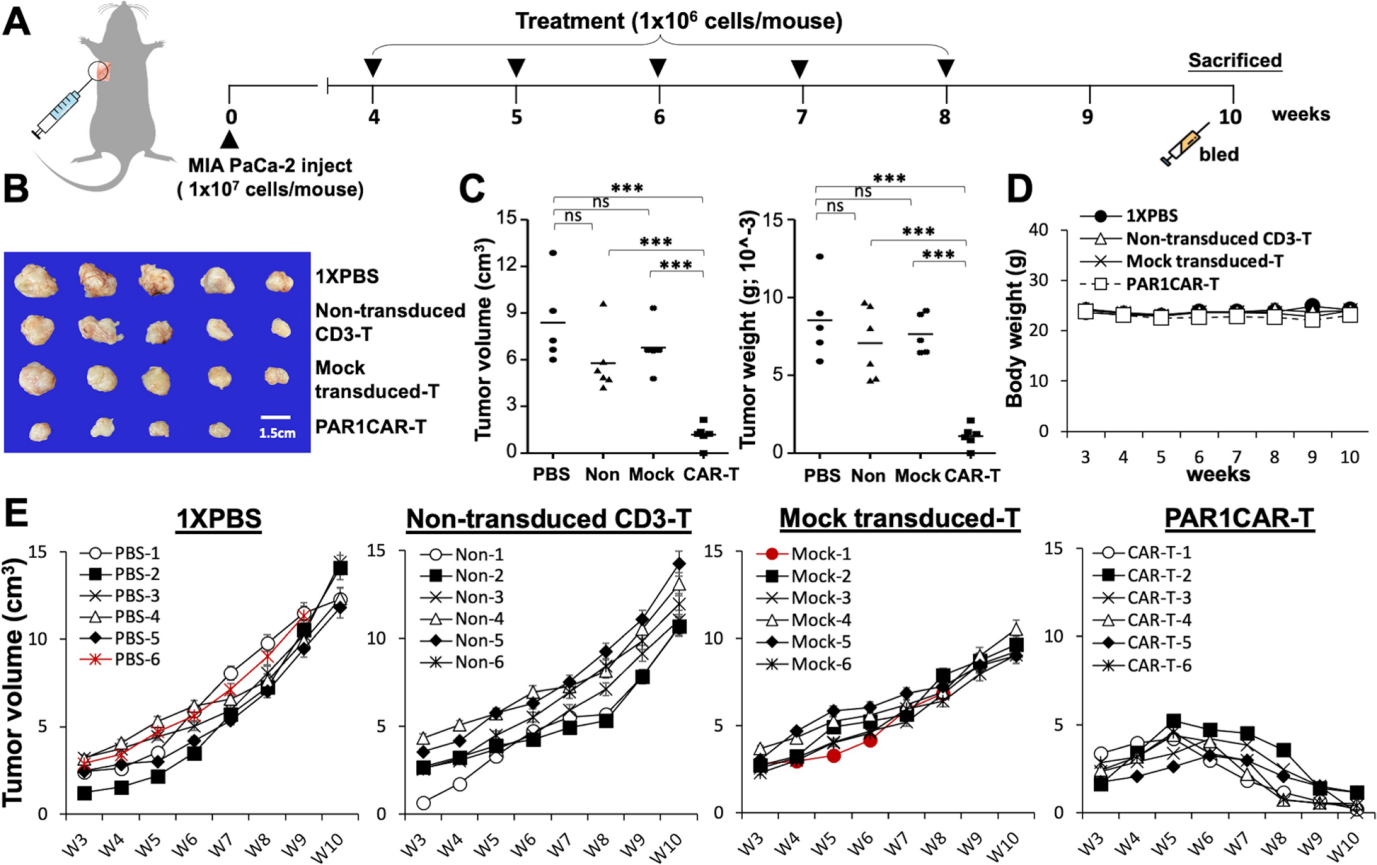
Predominant growth suppression on established subcutaneous MIA PaCa-2 xenografts by PAR1-targeted chimeric antigen receptor (CAR)-T cells in vivo*.* **A** Schematic representation of the detailed treatment schedule in the in vivo study. **B**, **C** Endpoint dissection of treated mice. **B** Photograph of a representative tumor mass. After dissection at week 10, tumor xenografts were first measured (the largest size was 2.9 cm) and then fixed with formalin. **C** For each treated mouse group, individual dissected tumor volumes (cm^3^) and individual dissected tumor weights (g) are expressed as scatterplots (*n* = 6 per group; *** *p* < 0.001). **D** Growth curve of mean body weight (g) of each treated mouse group showed no significant difference over time, whereas **E** growth curves of individual MIA PaCa-2 xenografts treated with 1 × PBS or indicated T cells. At the endpoint (at week 10), residual tumors treated with PAR1CAR-T cells were significantly smaller than those in control groups (*n* = 6 per group; *** *p* < 0.001). Two independent experiments are shown with similar results, and mice that died during the experimental period are shown in red

Nude mice bearing established subcutaneous MIA PaCa-2 xenografts were used to examine in vivo antitumor activities of PAR1CAR-T cells toward PAR1-expressing tumors. As illustrated in Fig. 5A, MIA PaCa-2 cell line xenograft models involved s.c. implanting 10^7^ cancer cells into a mouse, and allowing the cells to grow into a discernible tumor mass (approximately 2 × 10^3^ mm^3^ in volume) by 4 weeks after injection. Treatments were initiated from week 5. To determine the antitumor efficacy, we divided experimental mice into four groups and administered (i) PAR1CAR-T cells, (ii) mock-transduced T cells, (iii) non-transduced CD3 T cells, and (iv) 1 × PBS once per week for the next 5 consecutive weeks (from weeks 5 to 10). PAR1CAR-T cells displayed a potent antitumor effect, whereas others failed to suppress tumor growth (Fig. 5B, E). We also observed a significant and consistent cessation of MIA PaCa-2 tumors in mice treated with PAR1CAR-T cells (Fig. 5E). Moreover, mice treated with PAR1CAR-T cells did not exhibit body weight changes compared to all other treatments, implying that they had a heightened anticancer effect without causing an additional metabolic burden or systemic toxicity (Fig. 5D). At the experimental endpoint (at week 10), all mice treated with PAR1CAR-T cells exhibited significantly shrunken tumor volumes (cm^3^; *** *p* < 0.001) and decreased tumor weights (g; *** *p* < 0.001; Fig. 5C), whereas all of the other control mice had larger tumor burdens. These results indicated that PAR1CAR-T cells exerted strong antitumor effects on MIA PaCa-2 tumor cells in vivo.

**Fig. 6:**
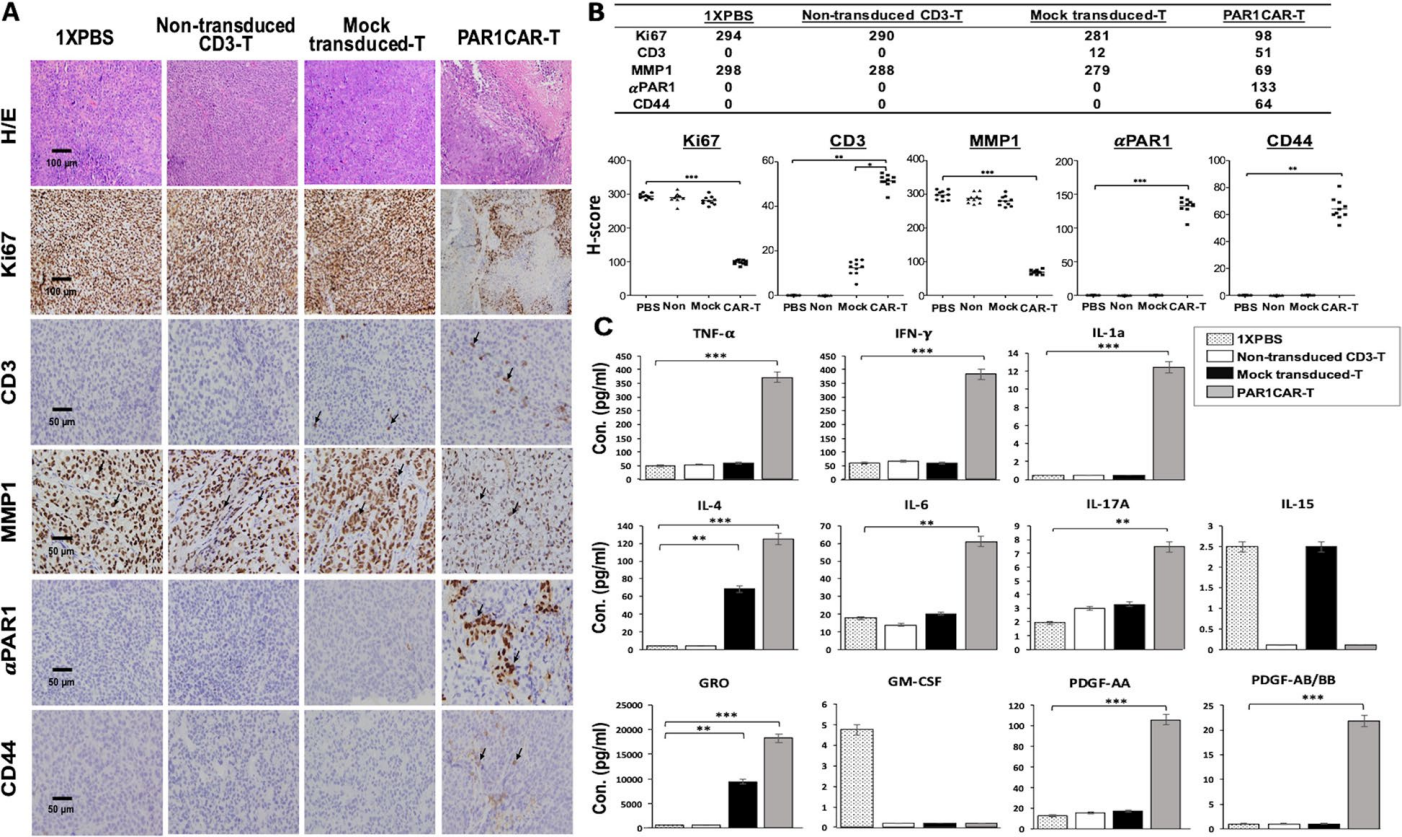
PAR1-targeted chimeric antigen receptor (CAR)-T cells were located in MIA PaCa-2 tumors. Tumors were collected from mice bearing MIA PaCa-2 subcutaneous xenografts treated with PAR1CAR-T cells, mock-transduced T cells, non-transduced cluster of differentiation 3-positive (CD3^+^) T cells, or 1 × PBS. **A** Formalin-fixed paraffin-embedded tumor sections were cut and stained with hematoxylin and eosin for human Ki67, CD3, matrix metalloproteinase (MMP)-1, anti-PAR1, and CD44 expressions (black arrowheads). Images were captured using a microscope (BX50; Olympus) and camera (DP22) at × 400 or × 200 original magnification. Individual scale bars are shown. **B** The average H-score for each marker and comparisons between groups are shown in scatterplots (* *p* < 0.05, ** *p* < 0.01, and *** *p* < 0.001). **C** Serum samples from each treated mouse group during the treatment period were subjected to a cytokine analysis using the Human Cytokine/Chemokine Magnetic Bead Panel protocol from Milliplex. Data presented are the concentrations (con., pg/mL) of selected proinflammatory cytokines/cytokines (tumor necrosis factor (TNF)-α, interferon (IFN)-γ, interleukin (IL)-1α, IL-4, IL-6, IL-17A, and IL-15; upper and middle panels), a chemokine (growth-related oncogene (GRO); bottom panel), and growth factors (granulocyte–macrophage colony-stimulating factor (GM-CSF), platelet-derived growth factor (PDGF)-AA, and PDGF-AB/BB; bottom panels). Cytokines/chemokines with undetectable levels in serum samples were excluded (data not shown). Data are presented as the mean ± SD of three independent experiments

The sustainable transfer of T cells in vivo is highly correlated with tumor regression [45]. We therefore examined the infiltration of human-modified T cells into tumor tissues in our established MIA PaCa-2 cell xenograft mice at the experimental endpoint. The persistence of human T cells was confirmed through immunostaining of sections of MIA PaCa-2 tumors treated with PAR1CAR-T cells. Results showed that human CD3^+^ T cells and αPAR1^+^ T cells had significantly accumulated and were retained in residual tumors after intravenous T-cell administration (Fig. 6A; * *p* < 0.05, ** *p* < 0.01, and *** *p* < 0.001), whereas no specific staining was detected in sections of tumors treated with mock-transduced T cells, non-transduced CD3 T cells, or 1 × PBS. We also detected tumor cell proliferation by Ki67 and MMP1 (a PAR1 ligand, also known as collagenase 1). MMP1 is often overexpressed by various cancers, as determined through MMP1-PAR1 signaling [46, 47] and staining of dissected tumor tissues. Tumor sections from control mice groups had much-higher Ki67 and MMP1 levels than those sections from genetically modified T-cell-treated mice (Fig. 6B; *** *p* < 0.001). Hematoxylin and eosin staining of tumor sections also confirmed that necrotic areas inside tumors were significantly larger in PAR1CAR-T-cell-treated tumor sections (Fig. 6A).

### Genetically engineered T cells secreted cytokines with enhanced antitumor functions in vivo

Activation of genetically modified T cells upon encountering antigens was accompanied by the release of cytokines and chemokines into the circulation. Serum samples from xenograft mice treated with PAR1CAR-T cells and control T cells, including non-transduced CD3 T cells, mock-transduced T cells (Mock), and 1 × PBS (Blank control), were collected to measure cytokine/chemokine levels using the human Cytokine/Chemokine Magnetic Bead Panel protocol from Milliplex (Fig. 6C). Among a total of 41 human cytokine/chemokine items available for inspection, 30 items revealed undetectable levels and were excluded from further statistical analysis (data not shown). The other 11 items were categorized into three major groups: proinflammatory cytokines (TNF-α, IFN-γ, IL-1α, IL-4, IL-6, and IL-17A), chemokines (GRO including CXCL1), and growth factors (GM-CSF, PDGF-AA, and PDGF-AB/BB).

Proinflammatory cytokines of TNF-α (*** *p* < 0.001), IFN-γ (*** *p* < 0.001), IL-1α (*** *p* < 0.001), IL-4 (*** *p* < 0.001), IL-6 (** *p* < 0.01), and IL-17A (** *p* < 0.01) in the serum of mice treated with PAR1CAR-T cells were significantly prolific, compared to the other three treatment groups (Fig. 6C, upper and middle panels). PAR1CAR-T cells encountering PAR1 tumor antigens led to significant increases in the levels of these cytokines. Higher serum chemokine GRO levels (*** *p* < 0.001), and levels of the growth factors PDGF-AA (*** *p* < 0.001), and PDGF-AB/BB (*** *p* < 0.001) in the PAR1CAR-T-cell-treated group were also observed than in the three other treatment groups (Fig. 6C, bottom panels). These data indicated that the substantial induction of systemic inflammatory cytokines was associated with CAR-T-cell activation following target antigen encounter, thereby enhancing the antitumor activity.

To further examine if PAR1CAR-T cells exerted cytotoxic activity against healthy tissues and healthy cell lines including MRC-5, WS1, and Hs181 cells, these cell lines were used as targets for in vitro cytolytic assays. Varying expressions of PAR1 from 1.2 to 7.8% in healthy cells were detected through flow cytometry (Additional file 3: Figure S3A). There was no significant cytolytic effect between individual treatments in all healthy cell lines (Additional file 3: Figure S3B). Regarding the concerns of toxicity of PAR1CAR-T cells against murine organs, including the heart, lungs, liver, kidneys, and testes, these organs were excised and histologically examined. Results also indicated that there were no significant morphological changes caused by off-target toxicity (Additional file 3: Figure S3C). Evaluation of selecting an optimal targeting threshold based upon four normal human cell lines, including MRC-5, WS1, Hs181.Tes, and Hs67, was proposed in diverse settings. Healthy cells were treated with various dosage titrations of TGF-β at 10, 30, and 60 ng/mL, and PAR1 expression was measured by flow cytometry at days 1, 3, and 7 following exposure, indicating both dosage- and time-dependent effects (Additional file 4: Figure S4A-D). Normal human cell lines were phenotyped by flow cytometry for PAR1 expression and showed representative quantified MFIs across samples (Additional file 4: Figure S4E). MTT assays were performed to measure standard 24-h cytotoxic activity in groups following PAR1CAR-T-cell therapy with different E/T ratios of 1, 5, and 20, and non-transduced CD3 T cell treatment, and mock-transduced T cell treatment against the four normal cell lines following 60 ng/mL TGF-β stimulation for 7 days (Additional file 4: Figure S4F). We conservatively assumed that if PAR1 MFI expression of cells or tissues was less than the threshold, then they were theoretically safe from being misidentified by PAR1CAR-T cells and sustaining damage.

### Relationship between TGF-β-modulated PAR1 and regulatory T (Treg) functions and tumor cell responses to PAR1CAR-T cell targeting

**Fig. 7:**
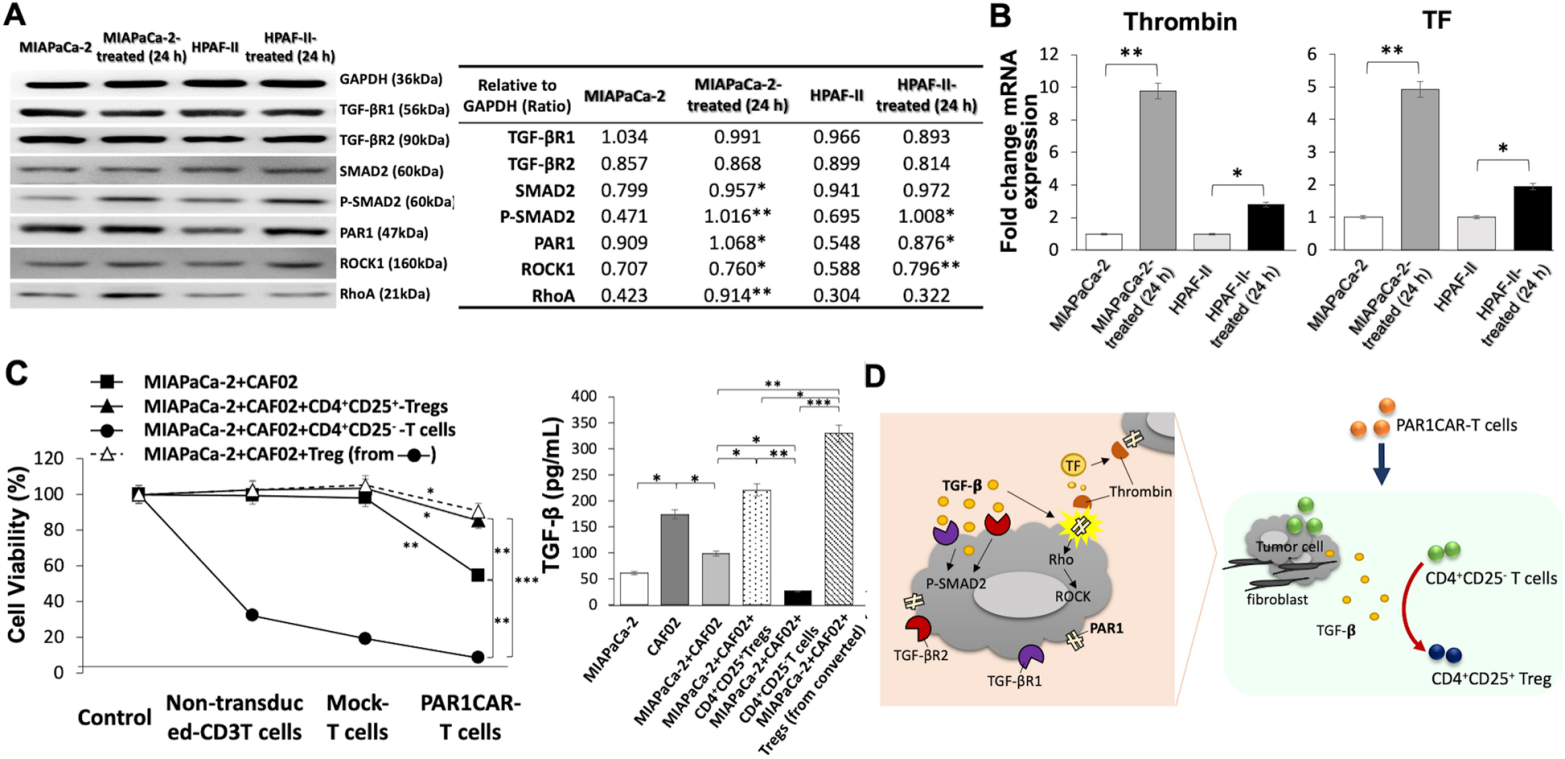
Relationship between transforming growth factor (TGF)-β-modulated PAR1 and regulatory T cell (Treg) function and pancreatic ductal adenocarcinoma (PDAC) cell response to PAR1CAR-T cell targeting. **A** Western blotting results of 24-h stimulation with TGF-β on the MIAPaCa-2 and HPAF-II PDAC cell lines. Glyceraldehyde-3-phosphate dehydrogenase (GAPDH) served as an internal control. **B** Analysis of tissue factor (TF) and thrombin expressions in individual cell lines treated for 24 h with the TGF-β growth factor. **C** Effect of adding cancer-associated fibroblasts (CAFs) and different phenotypes of cytokine-independent T cells on cell viability according to different treatments. Different co-culture combinations also resulted in various tumor-derived TGF-β levels. **D** A schematic diagram shows the role of immuno-mediated TGF-β affecting Treg function and transformation in PDAC treated with PAR1CART cells

Western blotting was used to measure type I TGF-β receptor (TβR-I), type II TGF-β receptor (TβR-II), and downstream Smads activation, as well as PAR1, ROCK1, and RhoA expressions in both MIAPaCa-2 and Capan1 cell lines. After 24 h of TGF-β stimulation, Smad2 was phosphorylated (*** *p* < 0.001 and ** *p* < 0.01) and PAR1 (*** *p* < 0.001 and ** *p* < 0.01), ROCK1 (** *p* < 0.01 and ** *p* < 0.01), and RhoA (*** *p* < 0.001 and ** *p* < 0.01) expressions were induced in MIAPaCa-2 and Capan1 cells. Nucleus translocation of Smad2 and phosphorylation of Smad2 through TGF-β signaling pathway activation were increased, as were molecules from a non-Smad-dependent pathway including RhoA from the Rho GTPase family and the Rho-associated kinase, ROCK1 (Fig. 7A).

We demonstrated that activation of TGF-β signaling regulates TF expression at the transcriptional level, thereby giving procoagulant characteristics to tumor cells that promoted tumor progression (Fig. 7B, right panel). As one of the most important ECM mediators, TGF-β demonstrates a pleiotropic immunoregulatory ability [48]. Of interest, it is widely recognized that thrombin stimulates the production of TGF-β, but we observed that TGF-β also regulated an increase in thrombin expression (Fig. 7B, left panel). TF is a transmembrane protein that can be expressed by various tumors. TF transforms its ligand factor VII into the active form, factor VIIa, and subsequently leads to a cancer-associated clotting cascade and also facilitates tumor growth through a coagulation-independent pathway [49, 50].

In human PDAC, Treg cells are defined as CD4^+^CD25^+^ T cells according to a previous study [51]. In the current study, PAR1CAR and CD4^+^CD25^−^ T cells were derived from a single donor. The effector (PAR1CAR-T cells) concentration-to-target (ET) ratios was set to 0.5 to treat PDAC cells (1.5 × 10^5^ cells) co-cultured with CAFs (1.5 × 10^5^ cells) with/without Treg cells (5 × 10^4^ cells), CD4^+^CD25^+^ Treg cells (5 × 10^4^ cells), and CD4^+^CD25^−^ T cells (5 × 10^4^ cells). Although the cytotoxic ability of PAR1CAR-T cells at the low ET ratio of 0.5 was relatively attenuated by adding CD4^+^CD25^+^ Treg cells, either original or newly converted ones, significant cytotoxic activities of PAR1CAR-T cells toward co-cultured tumor/CAF cells with or without additional Treg cells were observed, compared to the control, non-transduced CD3 T cells, and mock-transduced CD3 T cells (* *p* < 0.05 and ** *p* < 0.01; Fig. 7C, left panel). Various levels of derived TGF-β were observed in a sequential combination of cultures, with ELISAs demonstrating that MIAPaCa-2 + CAF02 + CD4^+^CD25^+^ Treg cells (converted from CD4^+^CD25^−^ T cells) expressed significantly higher levels of TGF-β compared to MIAPaCa-2 + CAF02 + CD4^+^CD25^−^ Treg cells (* *p* < 0.05), MIAPaCa-2 + CAF02 (** *p* < 0.01), and MIAPaCa-2 + CAF02 + CD4^+^CD25^−^ T cells (*** *p* < 0.001) (Fig. 7C, right panel), which were more sensitive to PAR1CAR-T cells (Fig. 7C, left panel). We also provide a supplement of tumor-labeled measurements to support direct tumor cell death using green fluorescent protein (GFP) fluorescence images (Additional file 5: Figure S5). Following 24-h treatment, significant cytotoxicity ability of PAR1CAR-T cells (at an E/T ratio of 0.5) toward GFP-labeled MIA PaCa-2 cells was detected in co-cultured conditions including CAFs (**p < 0.01) and CAFs plus CD4^+^CD25^+^ Treg cells (* *p* < 0.05), compared to control mock-transduced-T cells. Results are compatible with MTT assays (Fig. 7C).

A schematic diagram showed TGF-β modulable PAR1 remains a good target despite the ability of CD4^+^CD25^+^ Treg cells to suppress PAR1CAR-T cell action through a cell-contact mechanism. Treg cells induced CD4^+^CD25^−^ T cells to convert into new CD4^+^CD25^+^ Treg cells in the presence of a TGF-β secreted co-culture condition. TME-derived TGF-β also had an impact on antigen recognition and T cell activation to suppress antitumor immunity (Fig. 7D).

## Discussion

PDAC is an aggressive disease with unfavorable prognoses despite improvements in multimodality therapy thus far, and additional novel therapeutic targets and candidate molecules to escalate the treatment response are urgently needed. There are major barriers to applying CAR-T therapy for PDAC, including a lack of specific cancer-associated antigen expressions, complex logistics, an immune-suppressive TME, toxicity concerns, and manufacturing/financial restrictions. The role of immunotherapy in PDAC has yet to be determined.

PAR1 overexpression was found to be closely associated with tumor progression and poor survival outcomes in PDAC. Rather than being specific to tumor cells, PAR1 is expressed by the surrounding stroma that consists of endothelial cells, fibroblasts, and macrophages. Activation of stromal cell-associated PAR1 expression in the TME leads to increased vascular permeability, ECM production, and cytokine secretion, thereby promoting tumorigenesis [18, 52]. In this study, we developed PAR1-targeted CAR-T cells using third-generation CARs containing additional signaling domains, including CD28, CD137 (4-1BB), and CD3ζ (CD247), to augment activation of cytokine production and a tumor-eradication ability [38, 53]. PAR1-targeted CAR-T cells demonstrated specific killing potency both in vitro and in a xenograft murine model, accompanied by cytokine release.

Our analyses revealed that the cytotoxic activity of PAR1CAR-T cells toward PDAC cells was significantly correlated with the targeting specificity. Furthermore, in our cell line xenograft murine model, compared to mice treated with mock-transduced T cells, non-transduced CD3^+^ T cells, or 1 × PBS, PAR1CAR-T-cell-treated mice had significantly greater TME infiltration, cytokine and chemokine induction, and tumor-eliminating effects. The engineered CAR-T-cell affinity and efficacy were affected by the PAR1 antigen density on target cells in PDAC cell lines and the xenograft animal model. In the current study, we not only examined the influence of CAR affinity and antigen density on primary T cell activation but also its cytotoxic ability in vivo. A highly promising beginning was exhibited in the present study that suggests future applications of PAR1-targeted CAR-T-cell-based immunotherapy to human PDAC.

The level of clinical success when using CAR-transduced cells for PDAC treatment is limited because of the low immunogenicity of PDAC and recruitment of immunosuppressive cells from the TME that produce TGF-β. PAR1 expression was found to be strongly regulated by activation of latent-state TGF-β, a pleiotropic cytokine with dual roles of tumor suppression and tumor promotion in PDAC progression [4, 54]. Studies demonstrated that an elevated TGF-β level enhances PDAC progression [55], while a low level of TGF-β in circulation was associated with prolonged survival [56]. Furthermore, TGF-β acts as a multifunctional growth factor responsible for tissue homeostasis, and the process through which TGF-β signaling affects PAR1 expression is complex. (a) This process involves cell-type and integrin-specific expression (e.g., αvβ6 in epithelial cells and αvβ5 in fibroblasts) [44]. (b) Its regulation was reported to depend on Smad3, mitogen-activated protein kinase kinase (MEK), and extracellular signal-regulated kinase 1/2 (ERK1/2) signaling pathways [44, 57]. (c) TGF-β significantly increases PAR1 expression via the S1P/S1PR2/S1PR3 signaling pathway in regulating expressions of fibrosis- and epithelial-mesenchymal transition (EMT)-related proteins [58]. We found that TGF-β-mediated PAR1 upregulation is not only time but also tumor cell dosage dependent. This particularity increases recognition and cytotoxicity against tumor cells even if PAR1 expression is low or nil, and it also makes PAR1CAR-T-cell-specific targeting adequate. TGF-β was reported to regulate interactions between tumor cells and surrounding stroma [59]. Whether the combination of TGF-β and PAR1CAR-T therapy has better therapeutic efficacy compared to PAR1CAR-T monotherapy for PDAC, we have to accordingly discuss in vitro and in vivo points of view. In the current study, the cytotoxic ability of PAR1CAR-T therapy was clearly enhanced toward TGF-β-treated PAR1-expressing tumor cells in vitro. However, the intricate regulatory role of TGF-β-mediated PAR1 expression in response to PAR1CAR-T cell treatment in vivo requires further investigation. It is multifactorial and involves multiple tumoral and/or environmental factors in addition to the TGF-β paradox, such as the simulated extent of the TME, tumor differentiation, degree of surface antigen upregulation, Treg immunomodulation, the concentration, frequency, and delivery systems for administration, length of treatment time of added TGF-β, etc.

Compared to the stringent control of PAR1 activation in normal tissues, PAR1 is constitutively overexpressed by cancer cells through activation of many downstream signaling cascades including the MMP1, signal transducer and activator of transcription 3 (STAT3), AKT, and VEGF pathways [6]. MMP1-mediated PAR1 activates transcription factor nuclear factor (NF)-κB, which is known to promote PDAC cell invasion and angiogenesis [6, 60, 61]. MMP1 belongs to the metzincin protease superfamily, and it can degrade ECM components containing type I, II, III, VII, and X collagens, leading to antitumorigenic effects; MMP1 levels accordingly decline [62]. We showed that MMP1 levels, as surrogate markers for the response to treatment, in PAR1CAR-T-cell-treated tumor sections were significantly oriented downward compared to those treated with mock-transduced T cells, non-transduced T cells, and PBS. Our results indicated that the anti-tumorigenicity of PDAC cells was facilitated by PAR1-MMP1 signaling.

MMP1 was identified as an agonist against PAR1 [7]. To our knowledge, MMP1 expression in pancreatic cancer therapy has not been verified in Treg cells. Endothelial PAR1 is a non-tumor cell/non-matrix target of MMP1 produced by carcinoma cells. Activation of endothelial PAR1 by MMP1 enhanced endothelial permeability resulting in transendothelial migration [63]. Treg cells are able to increase MMP-1 expression in fibroblasts [64]. Research regarding wound healing also found that Treg-conditioned media stimulated the EMT, which led to E-cadherin downregulation. Treg cells also increased MMP1, which is involved in tissue remodeling [65]. High MMP1 expression was associated with a poor prognosis and immune cell infiltration, including Treg cells, in head and neck squamous cell carcinoma [66]. In the current study, MMP1 expression by tumors collected from mice bearing MIA PaCa-2 subcutaneous xenografts treated with PAR1CAR-T cells, mock-transduced T cells, non-transduced CD3^+^ T cells, and PBS control groups was demonstrated by IHC staining. MMP1 expression was significantly lower in the PAR1CAR-T-cell-treated group, compared to other groups. MMP1 is mainly located in tumoral stroma. In the process of effective PAR1CAR-T therapy, targeted tumor cells and CAFs were eliminated, and simultaneously, the MMP1-enriched ECM was degraded, which therefore led to loss of MMP1.

PAR1CAR-T cells secrete proinflammatory cytokines and chemokines, including TNF-α, IFN-γ, IL-1α, IL-4, IL-6, IL-17A, and GRO, upon encountering PAR1 antigens that exhibit potent cytolytic capacities against PAR1-expressing tumor cells. In accordance with complex inflammatory cytokine secretion profiles in mice serum, IL-17A elevation through increased helper T (Th)17 cell differentiation may involve contributions from TGF-β, IL-1, and IL-6 [57]. Mechanistically, IL-6 in combination with TGF-β leads to activation of STAT3 and retinoic-acid-receptor-related orphan nuclear receptor γt, thereby enhancing Th17 differentiation and IL-17A production [67]. Additionally, IL-1 promotes Th17 differentiation by downregulating TGF-β-induced Foxp3 expression [68]. Furthermore, the PDGF family comprises two linked chains that can be assembled as a heterodimer (PDGF-AB) or as homodimers (PDGF-AA, PDGF-BB, PDGF-CC, and PDGF-DD) and are expressed by various cell types, including activated platelets, macrophages, endothelial cells, osteoclasts, and tumor cells in the TME [69]. McCarty et al. reported that PDGF-BB upregulation inhibits PDAC growth by enhancing tumor pericyte recruitment [70]. However, the molecular role of PDGF in tumorigenesis remains obscure and requires further investigation.

It is evident that CAFs directly hinder the T-cell receptor (TCR) and interfere with antigen recognition and T-cell activation in PDAC. CAF-derived TGF-β also has a negative role in antigen-specific activation of specific CD8^+^ T cells [71]. In addition, CAF-derived inhibitory cytokines and TGF-β are required for Treg immunomodulation in PDAC. It seems that TGF-β not only mediates Treg functions but also induces tumor antigen tolerance, both of which lead to suppression of anticancer immunity [72]; however, the precise mechanism of suppression is not yet understood. Increasing evidence suggests that the Treg frequency is associated with tumor aggressiveness and clinical outcomes in PDAC [73, 74]. CD4^+^CD25^+^ Treg cells suppress cytotoxic T cells through a cell-contact mechanism of action [75]. Treg depletion showed an increased effect on the antitumor response of CD8^+^ T cells in an animal PDAC model [76]. Moreover, the sustained renewal of CD4^+^ T cells develops suppressive activity by either their nature or an educated consequence [77]. In the current study, we demonstrated that PAR1 remained an effective target of CAR-T cells toward tumor cells despite high TGF-β levels and TGF-β-mediated Treg cells in the immunosuppressive TME. On the other hand, limited evidence regarding the functional link between thrombin-PAR1 and TGF-β signaling pathways has been documented, and it was suggested that thrombin activates PAR1 on the endothelium and is important in angiogenesis and promoting tumor growth and invasion [78].

The accompanying release of multiple proinflammatory cytokines and chemokines from CAR-T cells targeting PAR1 enhances cytotoxicity against tumor cells. Most normal human tissues exhibit relatively low PAR1 expression except that lymphoid tissues revealed moderate PAR1 expression [79] (https://www.proteinatlas.org/ENSG00000181104-F2R). In our study, PAR1CAR-T therapy was harmless toward healthy PAR1^+^ cell lines in vitro and also showed negligible on-target off-tumor effects in our cell line xenograft murine model. A possible explanation is that the PAR1 antigen is mainly located intracellularly in normal tissues, compared to the cellular surface of some tumor cells. In addition, in healthy tissues, PAR1 signaling by T cells regulates adaptive immune responses and decreases damage to body cells. As our study shows, Treg cells play an important role in maintaining immune tolerance and weakening the cytotoxic ability of PAR1CAR-T-cells. TME-derived TGF-β upregulates PAR1 expression on the tumor surface and enhances antigen recognition and CAR-T-cell activation, thereby partially counteracting Treg immunosuppression. Collectively, it seems that PAR1 is a potential TAA in pancreatic cancer CAR-T-cell therapy because of the disparity between healthy and certain targeted malignant tissues. As CAR-T-cell technology matures, its use and translational intent will expand. In addition to current management strategies for alleviating adverse events (e.g., cytokine release syndrome and immune effector cell-associated neurotoxicity syndrome) related to CAR-T-cell therapy, including continuous monitoring, rapid detection, and accurate intervention with supportive care, anti-cytokine, and concurrent corticosteroid therapy are available for clinical use [80, 81].

This study has some limitations that need to be addressed. This study showed that PAR1-redirected CAR-T-cell therapy is a promising treatment strategy for PDAC both in vitro and in vivo; however, some issues need to be clarified before its clinical application. Future clinical trials are required to investigate the clinical and systemic toxicity, the optimal population to receive therapy, and the correlated efficacy in humans.

## Conclusions

In the present study, the efficacy of our PAR1-targeted CAR-T-cell treatment for PDAC both in vitro and in a xenograft mice model was significantly associated with targeting specificity. Our findings provide support for the use of PAR1-aimed CAR-T cells for targeted tumor elimination, and this strategy may improve future clinical PDAC treatment.

### Supplementary Information

**Additional file 1:** **Figure S1.** Characterization of genetically engineered PAR1CAR-T cells. αPAR1-specific chimeric antigen receptor (CAR) expression levels by human T cells transduced with lentiviral particles were analyzed using recombinant PAR1-His.Tag followed by flow cytometric antibody APC-anti-His.Tag conjugation for detecting αPAR1 expression. Transduction efficiencies are shown inside each panel.

**Additional file 2:** **Figure S2.** Enhanced specific suppression of PAR1-upregulated PaC cells by PAR1CAR-T cells in vitro. (A) Six human PaC cell lines were exposed to 1 ng/mL transforming growth factor (TGF)-β, and cells were collected at indicated times over the course of 48 h. PAR1 expression was measured by flow cytometry. Results showed the original and enhanced levels of PAR1 by the mean fluorescence intensity (MFI; left panel), expressed as fold-changes (right panel), as well as cell fold-changes (middle panel) over incubation times. (B~D) Standard 24-h cytotoxicity activities were performed using MTT assays with at least three replicates (*n* ≥ 3) with increasing effector/tumor (E/T) ratios of 0, 0.1, 1, 5, 10, and 20 against (B) HPAF-II, (C) CFPAC-1, and (D) MIA PaCa-2 cells following 1 ng/mL TGF-β stimulation for 48 h. Cytotoxic activities were compared with non-transduced CD3 T cell-treated cells and mock-transduced T cell-treated cells which served as controls of PAR1CAR-T cells (*n* ≥ 3; ** *p* < 0.01, *** *p* < 0.001, respectively). Results shown are the mean ± SD of three independent experiments.

**Additional file 3:** **Figure S3**. Safety evaluation of chimeric antigen receptor (CAR)-T cell therapy. (A) Flow cytometry revealing surface PAR1 levels in different normal human cell lines. (B) PAR1CAR-T cells exhibited no cytolytic activity against healthy MRC-5, WS1, and Hs181.Tes cells. Data are presented as the mean ± SD of three independent experiments. (C) Hematoxylin and eosin staining revealed no obvious off-target toxicity against major mice organs. Original magnification = 200×. Scale bars = 100 μm.

**Additional file 4:** **Figure S4**. Evaluation of the targeting threshold using PAR1CAR-T cell treatment. Four normal human cell lines, (A) MRC-5, (B) WS1, (C) Hs181.Tes, and (D) Hs67, were exposed to different dosages (ng/mL) of transforming growth factor (TGF)-β at 10, 30, and 60 ng/mL[?], and cells were collected on days 1, 3, and 7 following treatment. PAR1 expression was measured by flow cytometry. (E) Results shown are the potential enhanced levels of PAR1 by the mean fluorescence intensity (MFI) over incubation times. (F) Standard 24-h cytotoxicity activities were determined using MTT assays with at least three replicates (*n* ≥ 3) with increasing effector/tumor (E/T) ratios of 1, 5, and 20 against the four normal cell lines following 60 ng/mL TGF-β stimulation for 7 days. Cytotoxic activities were compared with non-transduced CD3 T cell-treated and mock-transduced T cell-treated groups which served as the controls of PAR1CAR-T cells (* *p* < 0.05). Results shown are the mean ± SD of three independent experiments.

**Additional file 5:** **Figure S5**. Significant cytotoxicity activities of PAR1CAR-T cells toward MIA PaCa-2 cells in different co-culture conditions by green fluorescent protein (GFP) labeling. GFP-labeled MIA PaCa-2 cells (MIA PaCa-2-GFP) co-cultured in different conditions including cancer-associated fibroblasts (CAFs), CAFs+CD4+CD25+regulatory T cells (Tregs), and CAF+CD4+CD25-T effector cells. Cell viability of cancer cells in response to PAR1CAR-T cells at an effector/tumor (E/T) ratio of 0.5 showed significant cytotoxicity activities toward MIA PaCa-2-GFP+CAFs (** *p* < 0.01; right panel) and MIA PaCa-2-GFP+CAFs+CD4+CD25+Tregs (* *p* < 0.05; right panel) compared to that of mock-transduced-T-cell controls following 24 h of treatment using GFP fluorescence imaging (left panel). The scale bar denotes 50 µm.

**Additional file 6.** Uncropped gels/blots images.

## Data Availability

The RT-qPCR, flow cytometric, and other study data will be shared upon reasonable request.

## Abbreviations

Ab: Antibody
asTF: Alternatively spliced tissue factor
CAF: Cancer-associated fibroblast
CAM: Cell adhesion molecule
CAR: Chimeric antigen receptor
CEA: Carcinoembryonic antigen
CI: Cell index
Ct: Crossing threshold
DAPI: 4,6-Diamidino-2-phenylindole
E/T: Effector/tumor
ECL: Enhanced chemiluminescent
EGF: Epidermal growth factor
ELISA: Enzyme-linked immunosorbent assay
FGF: Fibroblast growth factor
G-CSF: Granulocyte colony-stimulating factor
GM-CSF: Granulocyte-macrophage colony-stimulating factor
GRO: Growth-regulated oncogene
HER2: Human epidermal growth factor receptor 2
HIV: Human immunodeficiency virus
ICAM-1: Intercellular cell adhesion molecule-1
IFN: Interferon
IL: Interleukin
LV: Lentiviral vector
mAb: Monoclonal antibody
MCP: Monocyte chemotactic protein
MHC: Major histocompatibility complex
MIP: Macrophage inflammatory protein
MMP: Matrix metalloproteinase
MOI: Multiplicity of infection
MSN: Mesothelin
MTT: 3-(4,5-Dimethylthiazol-2-yl)-2,5-diphenyltetrazolium bromide
MUC1: Mucin 1
PAR: Protease-activated receptor
PBMC: Peripheral blood mononuclear cell
PBS: Phosphate-buffered saline
PDAC: Pancreatic ductal adenocarcinoma
PDGF: Platelet-derived growth factor
PE: Phycoerythrin
PROM1: Prominin 1
qPCR: Quantitative polymerase chain reaction
RANTES: Regulated upon activation normal T cell expressed and secreted
RT: Reverse transcription
SD: Standard deviation
STAT3: Signal transducer and activator of transcription 3
TAA: Tumor-associated antigen
TAM: Tumor-associated macrophage
TCR: T-cell receptor
TF: Tissue factor
TGF: Transforming growth factor
Th: Helper T
TM: Transmembrane
TME: Tumor microenvironment
TNF: Tumor necrosis factor
Treg: Regulatory T cell
TβR: TGF-β receptor
VCAM-1: Vascular cell adhesion protein-1
VEGF: Vascular endothelial growth factor
