# Supplementary material for: Effect of chimeric antigen receptor T cells against protease-activated receptor 1 for treating pancreatic cancer
Source: BMC Med. 2023 Sep 4;21:338. doi: 10.1186/s12916-023-03053-9 (PMC10478223; doi:10.1186/s12916-023-03053-9)
Supplement: Supplementary file 1 — Additional file 1: Figure S1. Characterization of genetically engineered PAR1CAR-T cells. αPAR1-specific chimeric antigen receptor (CAR) expression levels by human T cells transduced with lentiviral particles were analyzed using recombinant PAR1-His.Tag followed by flow cytometric antibody APC-anti-His.Tag conjugation for detecting αPAR1 expression. Transduction efficiencies are shown inside each panel. [file 12916_2023_3053_MOESM1_ESM.pdf]

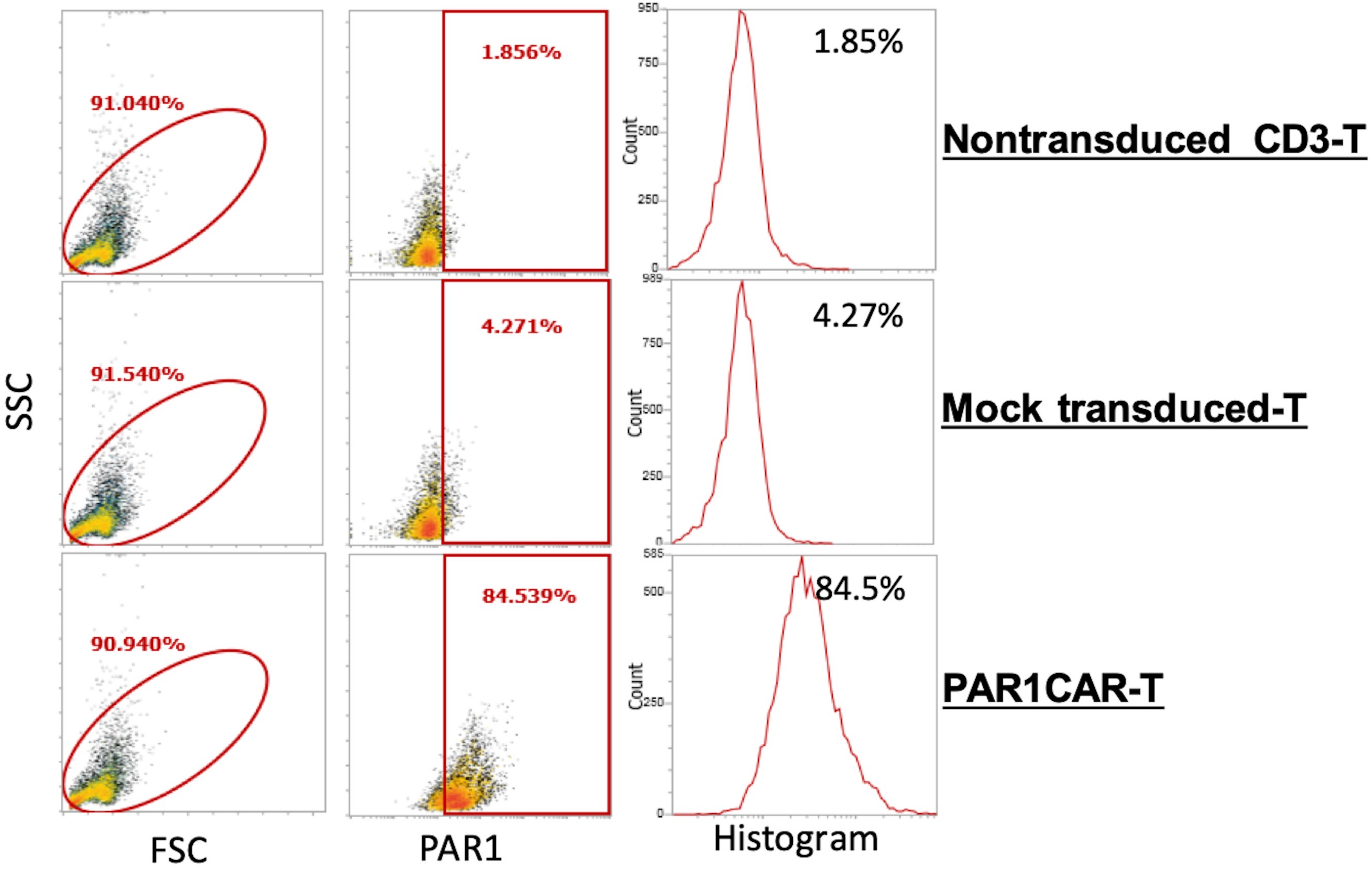

Figure S1. Characterization of genetically engineered PAR1CAR T cells. αPAR1-specific CAR (PAR1CAR) expression levels on human T cells transduced with lentiviral particles were analyzed using recombinant PAR1-His.Tag followed by flow cytometric antibody APC-anti-His.Tag conjugated for detecting αPAR1 expression. Transduction efficiencies are shown inside each panel.
