# Supplementary material for: Effect of chimeric antigen receptor T cells against protease-activated receptor 1 for treating pancreatic cancer
Source: BMC Med. 2023 Sep 4;21:338. doi: 10.1186/s12916-023-03053-9 (PMC10478223; doi:10.1186/s12916-023-03053-9)
Supplement: Supplementary file 2 — Additional file 2: Figure S2. Enhanced specific suppression of PAR1-upregulated PaC cells by PAR1CAR-T cells in vitro. (A) Six human PaC cell lines were exposed to 1 ng/mL transforming growth factor (TGF)-β, and cells were collected at indicated times over the course of 48 h. PAR1 expression was measured by flow cytometry. Results showed the original and enhanced levels of PAR1 by the mean fluorescence intensity (MFI; left panel), expressed as fold-changes (right panel), as well as cell fold-changes (middle panel) over incubation times. (B~D) Standard 24-h cytotoxicity activities were performed using MTT assays with at least three replicates (n ≥ 3) with increasing effector/tumor (E/T) ratios of 0, 0.1, 1, 5, 10, and 20 against (B) HPAF-II, (C) CFPAC-1, and (D) MIA PaCa-2 cells following 1 ng/mL TGF-β stimulation for 48 h. Cytotoxic activities were compared with non-transduced CD3 T cell-treated cells and mock-transduced T cell-treated cells which served as controls of PAR1CAR-T cells (n ≥ 3; ** p < 0.01, *** p < 0.001, respectively). Results shown are the mean ± SD of three independent experiments. [file 12916_2023_3053_MOESM2_ESM.pdf]

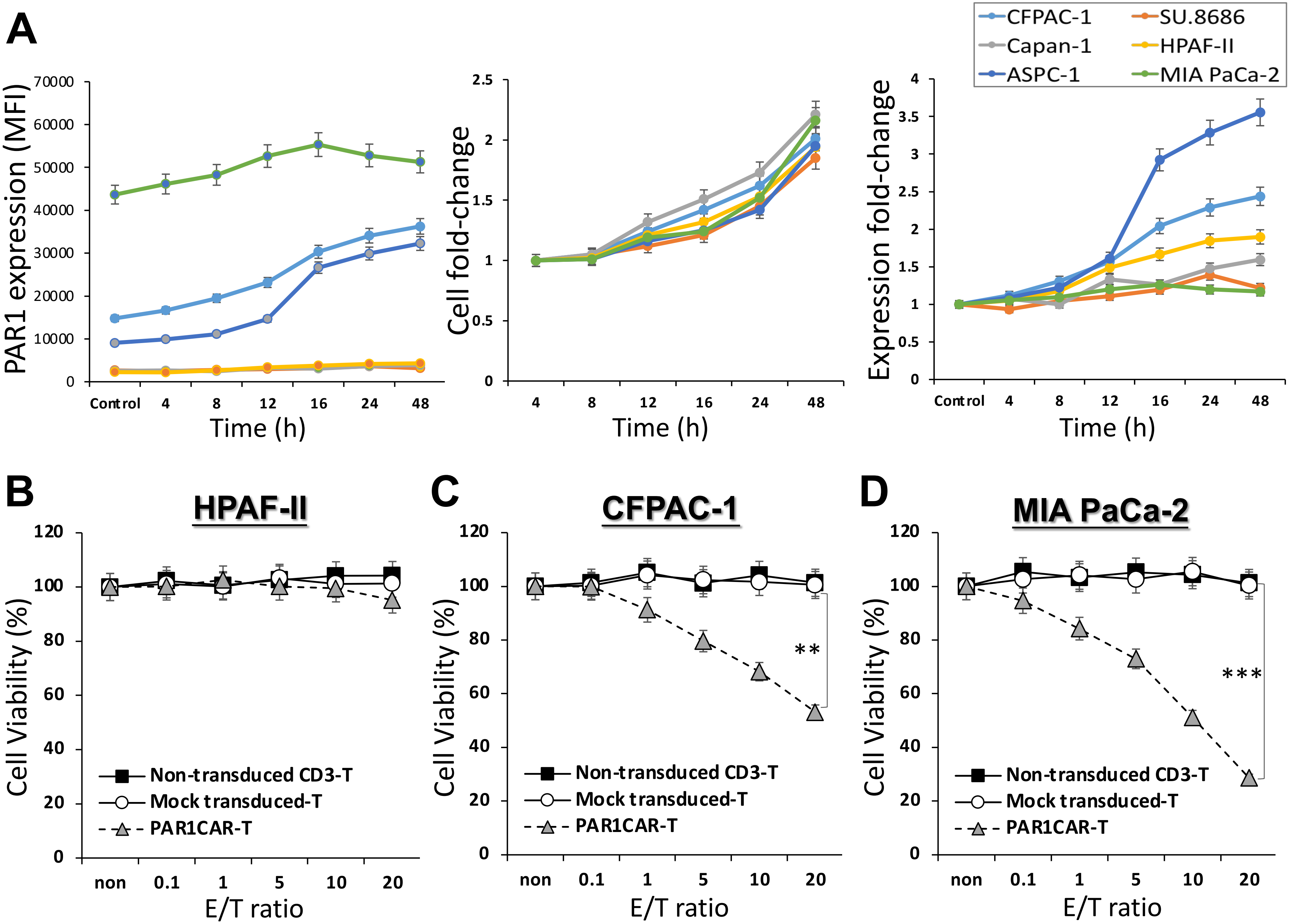

Figure S2: Enhanced specific suppression of PAR1-upregulating PaC cells by PAR1CAR-T cells *in vitro*. (A) Six human PaC cell lines were exposed to 1 ng/mL TGF$\boldsymbol{\beta}$ and cells collected at indicated times over the course of 48 hours. PAR1 expression was measured by flow cytometry. Results showed the original and enhanced levels of PAR1 by mean fluorescence intensity (MFI; left panel), expressed fold-change (right panel), as well as cell fold-change (middle panel) over incubation times**. (B~D)** Standard 24-h cytotoxicity activities were performed using MTT assays with at least three replicates (n > 3) with increasing E/T ratios of 0, 0.1, 1, 5, 10, and 20 against **(B)** HPAF-II, **(C)** CFPAC-1, and **(D)** MIA PaCa-2 cells following 1 ng/mL TGF**β**-stimulated for 48 hs. Cytotoxic activities were compared with nontransduced CD3 T cell-treated cells, and mock-transduced T cell-treated cells served as the controls of PAR1CAR-T cells (n > 3; ***p* < 0.01, ****p* < 0.001, respectively). Results shown are the mean ± SD of three independent experiments.
