# Supplementary material for: Effect of chimeric antigen receptor T cells against protease-activated receptor 1 for treating pancreatic cancer
Source: BMC Med. 2023 Sep 4;21:338. doi: 10.1186/s12916-023-03053-9 (PMC10478223; doi:10.1186/s12916-023-03053-9)
Supplement: Supplementary file 3 — Additional file 3: Figure S3. Safety evaluation of chimeric antigen receptor (CAR)-T cell therapy. (A) Flow cytometry revealing surface PAR1 levels in different normal human cell lines. (B) PAR1CAR-T cells exhibited no cytolytic activity against healthy MRC-5, WS1, and Hs181.Tes cells. Data are presented as the mean ± SD of three independent experiments. (C) Hematoxylin and eosin staining revealed no obvious off-target toxicity against major mice organs. Original magnification = 200×. Scale bars = 100 μm. [file 12916_2023_3053_MOESM3_ESM.pdf]

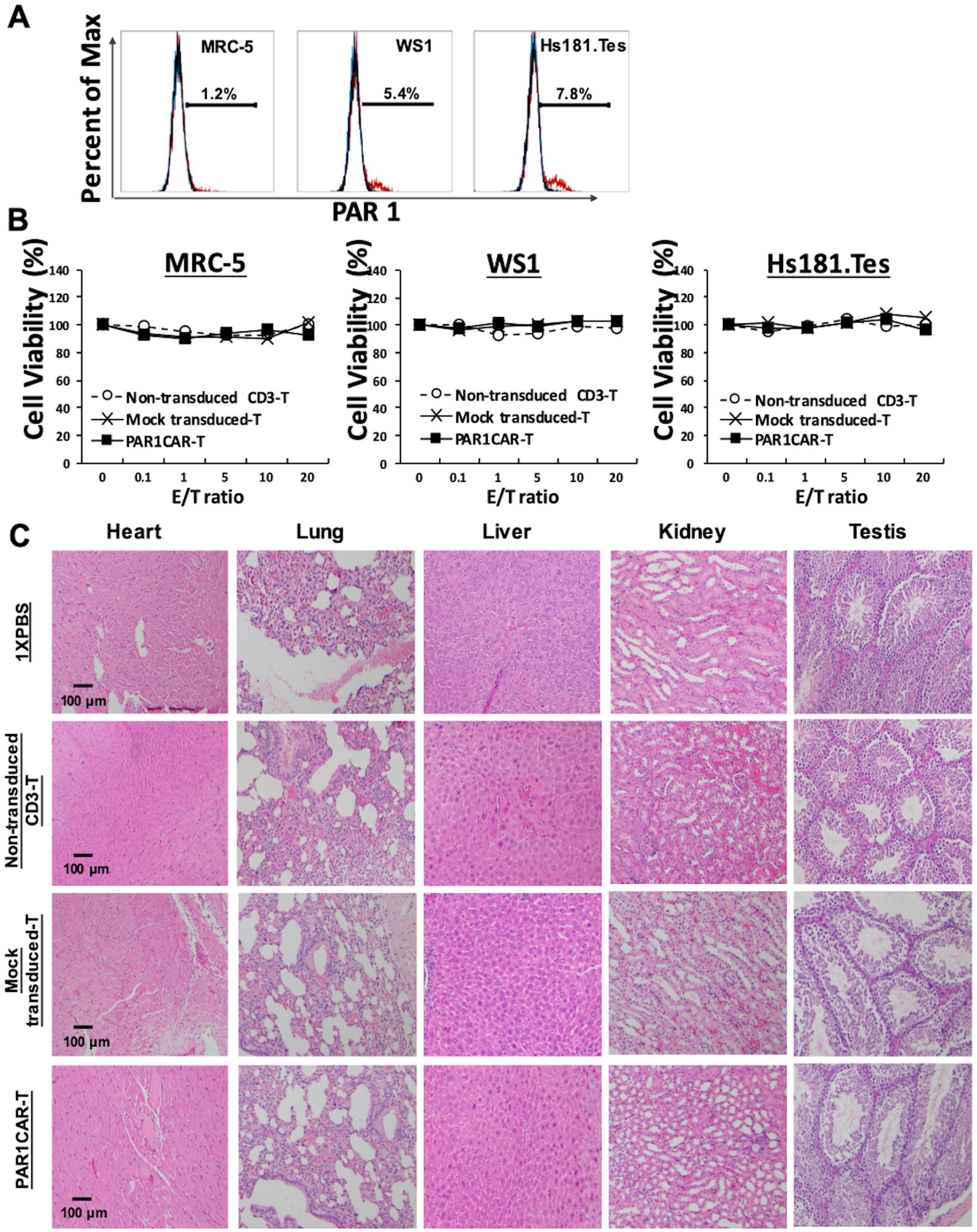

Figure S3. Safety Evaluation of CAR T Cell Therapy (A) Flow cytometry revealing surface PAR1 levels in different normal human cell lines. **(B)** PAR1CAR T cells exhibit no cytolytic activity against healthy MRC-5, WS1, and Hs181.Tes cells. Data are presented as the mean ± SD of three independent experiments. **(C)** Hematoxylin and eosin staining revealing no obvious off-target toxicity against major mice organs. Original magnification = 200 ×. Scale bars = 100 μm.
