# Supplementary material for: Effect of chimeric antigen receptor T cells against protease-activated receptor 1 for treating pancreatic cancer
Source: BMC Med. 2023 Sep 4;21:338. doi: 10.1186/s12916-023-03053-9 (PMC10478223; doi:10.1186/s12916-023-03053-9)
Supplement: Supplementary file 5 — Additional file 5: Figure S5. Significant cytotoxicity activities of PAR1CAR-T cells toward MIA PaCa-2 cells in different co-culture conditions by green fluorescent protein (GFP) labeling. GFP-labeled MIA PaCa-2 cells (MIA PaCa-2-GFP) co-cultured in different conditions including cancer-associated fibroblasts (CAFs), CAFs+CD4+CD25+regulatory T cells (Tregs), and CAF+CD4+CD25-T effector cells. Cell viability of cancer cells in response to PAR1CAR-T cells at an effector/tumor (E/T) ratio of 0.5 showed significant cytotoxicity activities toward MIA PaCa-2-GFP+CAFs (** p < 0.01; right panel) and MIA PaCa-2-GFP+CAFs+CD4+CD25+Tregs (* p < 0.05; right panel) compared to that of mock-transduced-T-cell controls following 24 h of treatment using GFP fluorescence imaging (left panel). The scale bar denotes 50 µm. [file 12916_2023_3053_MOESM5_ESM.pdf]

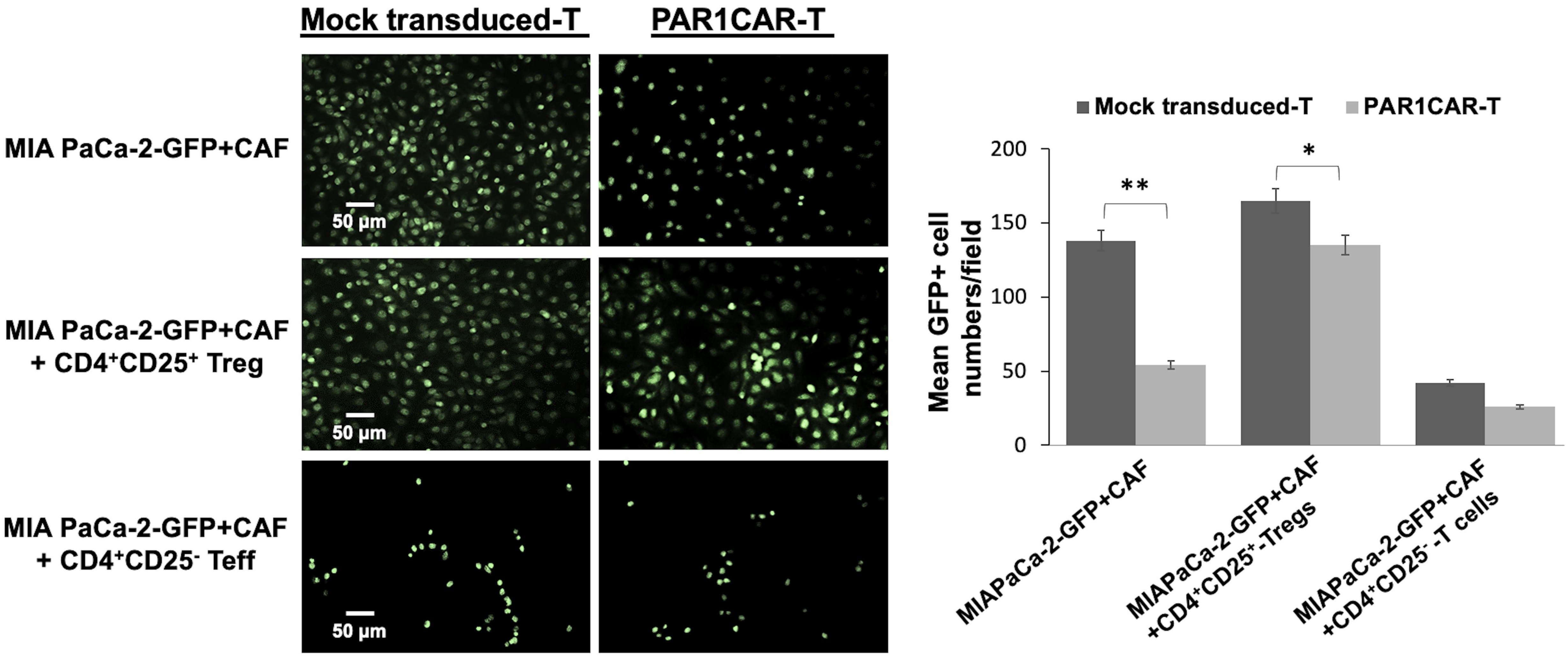

Figure S5. Significant cytotoxicity activities of PAR1CAR-T cells toward MIA PaCa-2 cells in different co-culture conditions by GFP labeling. The GFP-labeled MIA PaCa-2 cells (MIA PaCa-2-GFP) co-cultured with different conditions including CAF, CAF+$CD4^+CD25^+$Tregs, and CAF+CD4+CD25-T effector cells. Cell viability of cancer cells in response to PAR1CAR-T cells at E/T ratio of 0.5 showed significant cytotoxicity activities toward MIA PaCa-2-GFP+CAF (**$p < 0.01$; right panel), and MIA PaCa-2-GFP+CAF+$CD4^+CD25^+$Tregs (*$p < 0.05$; right panel) compared to that of mock-transduced-T cells control following 24 h treatment by using GFP fluorescence imaging (Left panel). The scale bar denotes 50 μm.
