# Supplementary material for: Effect of chimeric antigen receptor T cells against protease-activated receptor 1 for treating pancreatic cancer
Source: BMC Med. 2023 Sep 4;21:338. doi: 10.1186/s12916-023-03053-9 (PMC10478223; doi:10.1186/s12916-023-03053-9)
Supplement: Supplementary file 6 — Additional file 6. Uncropped gels/blots images. [file 12916_2023_3053_MOESM6_ESM.pdf]

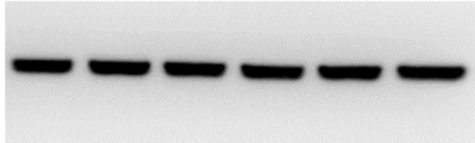

Figure 2B. GAPDH

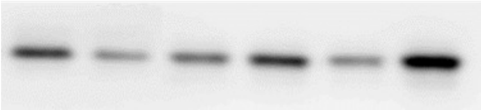

Figure 2B. PAR1

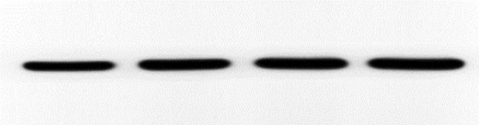

Figure 7A. GAPDH

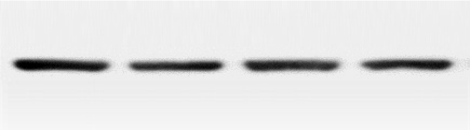

Figure 7A. TGF-βR1

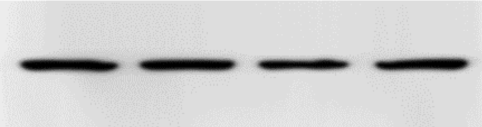

Figure 7A. TGF-βR2

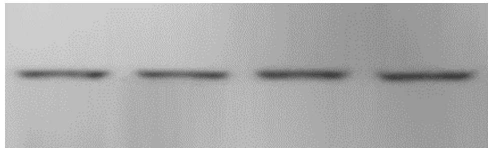

Figure 7A. SMAD2

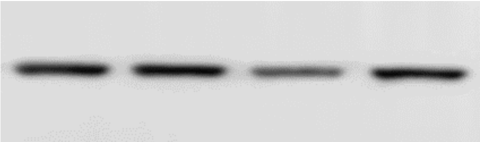

Figure 7A. PAR1

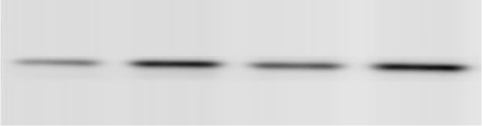

Figure 7A. P-SMAD2

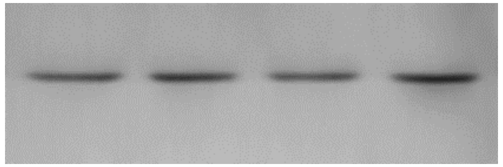

Figure 7A. ROCK1

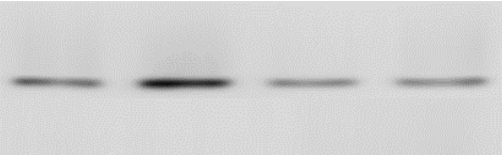

Figure 7A. RhoA
