# Supplementary material for: Effect of chimeric antigen receptor T cells against protease-activated receptor 1 for treating pancreatic cancer
Source: BMC Med. 2023 Sep 4;21:338. doi: 10.1186/s12916-023-03053-9 (PMC10478223; doi:10.1186/s12916-023-03053-9)
Supplement: Supplementary file 4 — Additional file 4: Figure S4. Evaluation of the targeting threshold using PAR1CAR-T cell treatment. Four normal human cell lines, (A) MRC-5, (B) WS1, (C) Hs181.Tes, and (D) Hs67, were exposed to different dosages (ng/mL) of transforming growth factor (TGF)-β at 10, 30, and 60 ng/mL[?], and cells were collected on days 1, 3, and 7 following treatment. PAR1 expression was measured by flow cytometry. (E) Results shown are the potential enhanced levels of PAR1 by the mean fluorescence intensity (MFI) over incubation times. (F) Standard 24-h cytotoxicity activities were determined using MTT assays with at least three replicates (n ≥ 3) with increasing effector/tumor (E/T) ratios of 1, 5, and 20 against the four normal cell lines following 60 ng/mL TGF-β stimulation for 7 days. Cytotoxic activities were compared with non-transduced CD3 T cell-treated and mock-transduced T cell-treated groups which served as the controls of PAR1CAR-T cells (* p < 0.05). Results shown are the mean ± SD of three independent experiments. [file 12916_2023_3053_MOESM4_ESM.pdf]

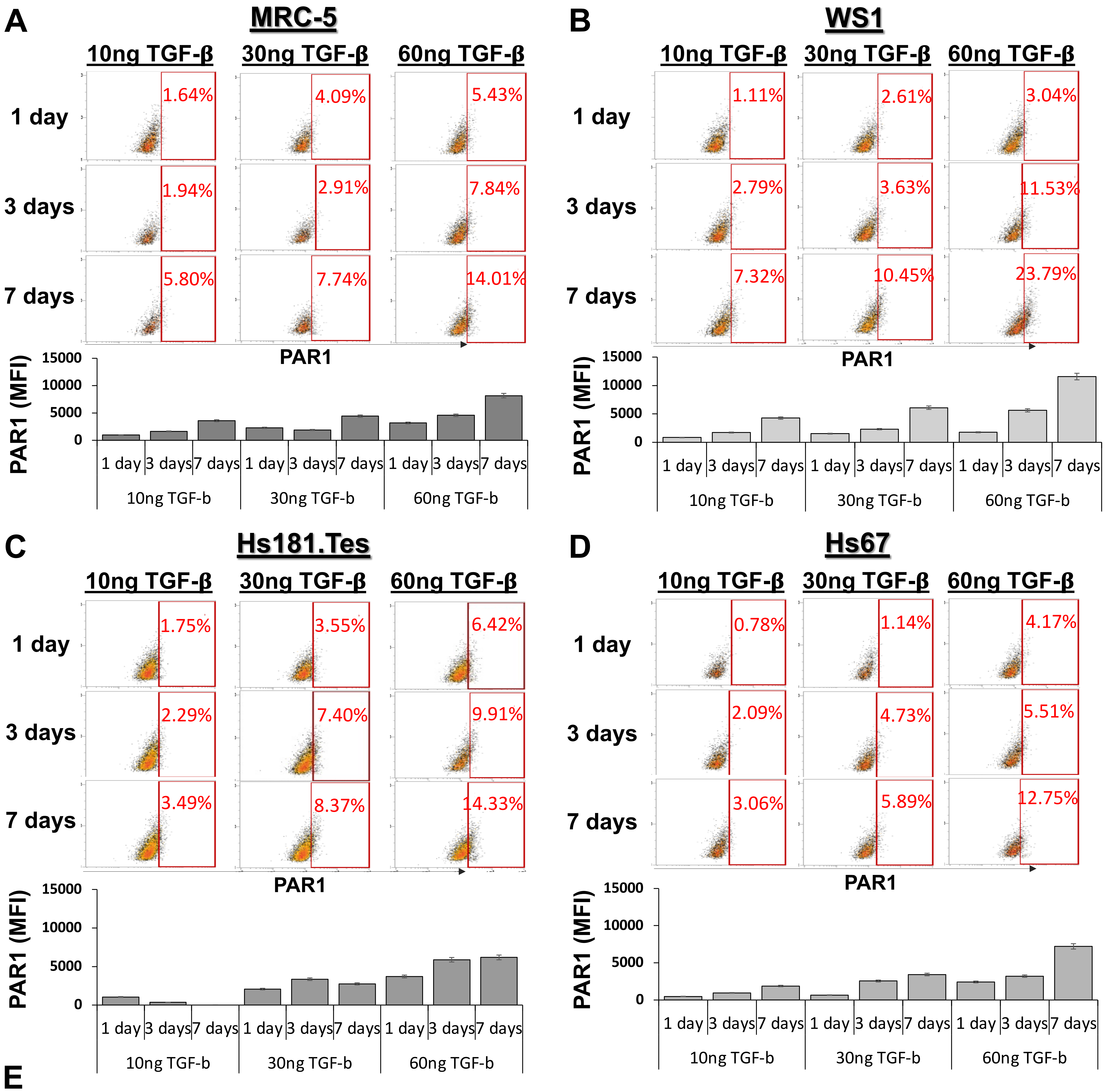

E

PAR1 Level (MFI)	Cell line	10ng/mL TGF-β			30ng/mL TGF-β			60ng/mL TGF-β		
		1 day	3 days	7 days	1 day	3 days	7 days	1 day	3 days	7 days
	WS1	844	1724	4268	1524	2317	6093	1770	5626	11574
	MRC-5	956	1631	3584	2283	1896	4416	3166	4571	8172
	Hs181.Tes	1052	352	0.00	2072	3356	2749	3705	5878	6178
	Hs67	487	959	1886	664	2555	3433	2429	3211	7214

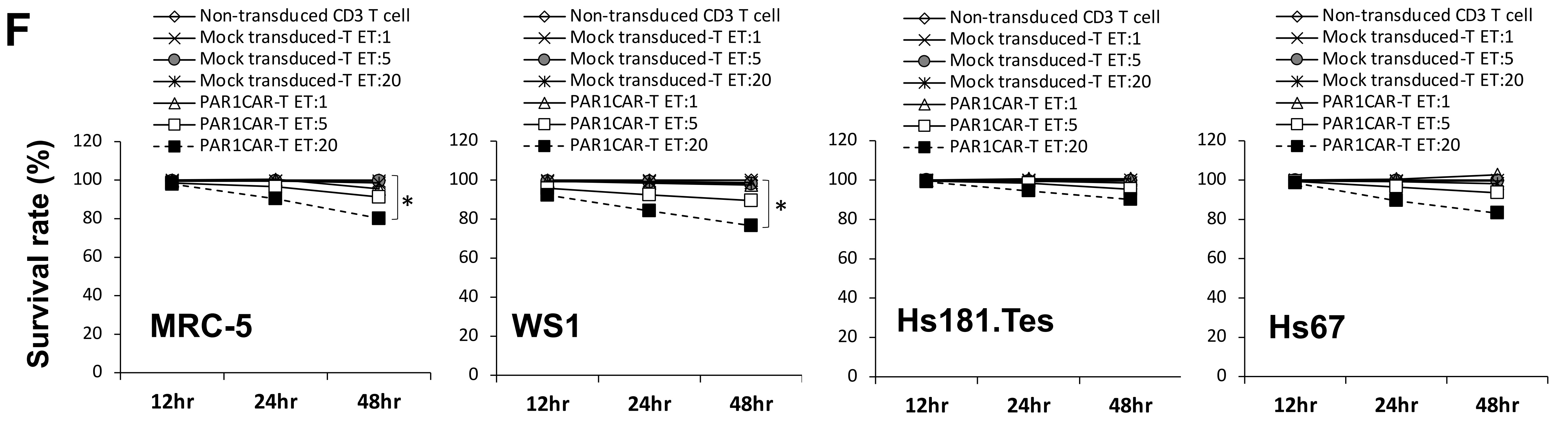

Figure S4. Evaluation of the Targeting Threshold by using PAR1CAR-T Cells Treatment. Four normal human cell lines **(A)** MRC-5, **(B)** WS1, **(C)** Hs181.Tes, and **(D)** Hs67 were exposed to different dosage (ng/mL) titration of TGF-**β** at 10, 30, and 60, and cells were collected at days 1, 3, and 7 following treatment periods. PAR1 expression was measured by flow cytometry. **(E)** Results shown are the potential enhanced levels of PAR1 by mean fluorescence intensity (MFI) over incubation times. **(F)** Standard 24-h cytotoxicity activities were performed using MTT assays with at least three replicates (n > 3) with increasing E/T ratios of 1, 5, and 20 against the four normal cell lines following 60 ng/mL TGF-**β** stimulated for 7 days. Cytotoxic activities were compared with non-transduced CD3 T cell-treated, mock-transduced T cell-treated which served as the controls of PAR1CAR-T cells (*$p < 0.05$). Results shown are the mean ± SD of three independent experiments.
